# Development of orthographic, phonological and semantic parafoveal processing in Chinese reading

**DOI:** 10.1177/17470218251372482

**Published:** 2025-08-22

**Authors:** Min Liu, Sainan Li, Zhu Meng, Yongsheng Wang, Chuanli Zang, Guoli Yan, Simon P Liversedge

**Affiliations:** 1 Key Research Base of Humanities and Social Sciences of the Ministry of Education, Tianjin Social Science Laboratory of Students’ Mental Development and Learning, Faculty of Psychology, Tianjin Normal University, Tianjin, China; 2 School of Educational Sciences, Liaocheng University, Liaocheng, China; 3 Institute of Moral Education and Educational Psychology, Tianjin Academy of Educational Sciences, Tianjin, China; 4 School of Educational Sciences, Jiangsu Normal University, Xuzhou, China; 5 School of Psychology, Liverpool Hope University, Liverpool, UK; 6 School of Psychology and Humanities, University of Lancashire, Preston, UK; 7 Northumbria University, Newcastle upon Tyne, UK

**Keywords:** Chinese reading development, preview benefit, parafoveal processing

## Abstract

Parafoveal pre-processing of upcoming words is a key aspect of fluent reading. A comparative analysis of how children’s orthographic, phonological and semantic parafoveal processing changes with age has not been investigated to date. In the present study, three eye movement experiments used the boundary paradigm to characterize the nature of change in orthographic, phonological and semantic parafoveal processing across children in Grades 2 to 5 (*n* = 366, Tianjin Primary School) and adults (*n* = 90, Tianjin Normal University) during natural Chinese reading. In each experiment we manipulated preview type (identical, related or unrelated preview). The results showed that effective orthographic parafoveal processing occurred in all our participant groups; however, effective phonological and semantic parafoveal processing was somewhat delayed, occurring in the third or fourth grade through to adults. We suggest that the differential developmental time course of orthographic relative to phonological and semantic parafoveal processing likely arises because the phonological and semantic characteristics of a written character are accessed via the character’s orthographic code. Orthographic parafoveal processing, therefore, likely takes developmental precedence over phonological and semantic parafoveal processing. Together, the results provide a quite comprehensive picture of how a fundamental aspect of reading, parafoveal processing, develops with age.

How children’s orthographic, phonological and semantic processing changes as they progress from early stages of reading to become more skilled readers remains a basic and fundamental question that is not fully understood. Furthermore, the nature and time course of such processing in relation to precise patterns of fixations made on words as they are read, and how these change with development, has been investigated only minimally. The present eye movement experiments, together, represent an examination of change in efficacy of orthographic, phonological and semantic parafoveal processing across the age range of 7 to 11 years. The development of effective and efficient parafoveal processing is a hallmark of skilled adult reading. In the present research, we wished to characterize the nature of change with age in these processes.

During reading, readers encode and process information about the word currently under fixation (i.e., via foveal processing), as well as the upcoming word to the right of fixation (i.e., via parafoveal processing) (see Rayner, 1998, 2009; Schotter et al., 2012). The acquisition of useful information from text to the right of the fixated word is often referred to as parafoveal preview, and this is achieved via parafoveal pre-processing (Rayner, 1975, 1998, 2009). In recent years there has been an increase in studies investigating children’s parafoveal pre-processing (see Johnson et al., 2018; Marx et al., 2015, 2016; Milledge et al., 2021, 2022; Pagán et al., 2016, 2021; Staroverova et al., 2023; Tiffin-Richards & Schroeder, 2015). The ability to parafoveally process upcoming words develops with reading skill (and under normal learning conditions, with age; see Häikiö et al., 2009; Rayner, 1986; Sperlich et al., 2015; Vorstius et al., 2014), and it is documented that as children’s reading develops, the extent of the perceptual span to the right of fixation from which useful information can be extracted and processed during reading increases.

Tiffin-Richards and Schroeder (2015) conducted a boundary paradigm experiment (Rayner, 1975) to investigate the type of information that German-speaking children (compared to adults) extract from parafoveal words. In this paradigm, an invisible boundary is located in a sentence immediately preceding a target word. Prior to the reader’s eyes crossing the boundary, a preview stimulus is presented in place of the target and the characteristics of the preview, in relation to those of the target, are manipulated. When the reader’s eyes cross the boundary, the preview is very rapidly replaced by the target and if the preview facilitates processing of the target (e.g., shorter fixation durations), this demonstrates preview benefit. Thus, the boundary paradigm allows us to investigate characteristics of a word that are processed whilst it is in the parafovea. When Tiffin-Richards and Schroeder used transposed letter previews that were orthographically related to the target, in children aged 7.9 to 9.1 years, preview benefit effects occurred only for the single fixation duration measure on the target word. However, the same stimuli in adults produced effects for first fixation, single fixation and gaze duration measures. That is to say, the effects were more robust and longer lasting, and arguably, earlier, in adults compared with children, suggesting that adult readers more effectively parafoveally process orthographic information than children.

Using the same paradigm, Pagán et al. (2016) manipulated the position of transposed letter pairs within the initial trigram of English words in parafoveal preview, and found that with increased age, children (aged: 8.1–9.6 years) can process parafoveal orthographic information even when the parafoveal letters of a word are transposed. Indeed, such flexible parafoveal orthographic processing occurred to a degree that was comparable to adults when the target word was directly fixated. This finding differs from that of Tiffin-Richards and Schroeder (2015). Pagán et al. explained this difference due to the child participants in their study having a reading age of 11.1 years on average (i.e., older than their chronological age), leading to more adult-like performance with respect to orthographic parafoveal processing.

Another study by Johnson et al. (2018) investigated the relationship between children’s age, their oral reading fluency and their decoding skills in relation to their parafoveal orthographic processing efficiency. Using the boundary paradigm, Johnson et al. manipulated predictability and parafoveal preview for target words embedded in sentences for developing readers aged from 6 to 12 years old. Target words appeared in predictable or neutral contexts and there were three preview types: identical (e.g., *apple*), visually similar (e.g., *apydo*), and a visually dissimilar preview (e.g., *egydo*). Predictability effects occurred, but more important for the present discussion, the results indicated that readers obtained partial letter identity information from an upcoming word and this sensitivity developed with age and reading fluency. Children with reading fluency at a second-grade level showed only numerical differences in orthographic preview benefits (a 6 ms difference between visually similar and visually dissimilar previews), while more fluent fifth or sixth grade readers showed statistically robust orthographic preview benefits (a 76-ms effect). Thus, Johnson et al.’s results support Pagán et al.’s suggestion that reading ability may modulate children’s parafoveal orthographic processing. To summarize, all of these studies, together, demonstrate that in alphabetic languages, children process orthographic information in the parafovea, though such processing is delayed relative to that in adults and the degree of delay is determined by reading ability.

To date, there have been only two published studies that have investigated children’s parafoveal phonological processing during natural reading.^1^ In the study by Tiffin-Richards and Schroeder (2015), described earlier, they also included stimuli in which the phonological characteristics of parafoveal stimuli were manipulated in relation to the phonological characteristics of the target. They included identical previews (e.g., *Blech*), pseudohomophone previews (e.g., *Bläch*) and control previews (e.g., *Blüch*). Tiffin-Richards and Schroeder demonstrated phonological preview benefit effects for children, but quite surprisingly, no such effects for the adults in their study (see also W. Zhou et al., 2018). Tiffin-Richards and Schroeder argued that children may depend to a greater degree on phonological processing relative to adults, and that this may have caused their pattern of effects. Recently, Milledge et al. (2022) have also shown that both adults and 8 to 9-year-old children are able to pre-lexically process phonology in the parafovea. Furthermore, such pre-processing is contingent on the degree of orthographic similarity between preview and target, with a greater advantage of phonological processing in orthographically similar previews. Overall, these studies indicate that even 8-year-old readers of English and German have developed the ability to process phonological information parafoveally. However, it is unclear what information can be extracted during Chinese reading in children.

Finally, as noted above, we also aimed to investigate semantic parafoveal processing, and we did this because the precise time course of the availability of semantic parafoveal information during reading is a topic of contention. This issue lies at the heart of a debate of significance in respect of the fundamental nature of reading, as well as in relation to different categories of formal computational models of eye movement control in reading; are words lexically identified for meaning serially and sequentially, or in parallel? Serial models of eye movement control (e.g., E-Z Reader, Reichle et al., 1998) stipulate that words are lexically processed serially, while parallel models (e.g., SWIFT, Engbert & Kliegl, 2011; OB1-Reader, Snell et al., 2018) assume that multiple words are lexically identified simultaneously, and potentially, in a non-sequential order. These contrasting positions lead to different theoretical predictions regarding the time course of the availability of semantic information from parafoveal words. Serial accounts specify that a word should not be identified until after it is fixated; that is, semantic parafoveal processing of words should not occur. In contrast, parallel accounts specify that semantic characteristics of parafoveal words should often become available prior to a word being fixated, and consequently, semantic parafoveal information should affect the duration of fixations on words when they are subsequently fixated. More recently, a further model of eye movement control that is relevant to the current article, has been developed. The Chinese Reading Model (CRM; Li & Pollatsek, 2020) is constructed around an Interactive Activation word identification system whereby parafoveal characters within the perceptual span activate lexical entries that are consistent with those characters. Oculomotor commitments (where and when the eyes move next) are then determined based on the lexical entry that is activated to the greatest degree and upcoming words are simultaneously lexically identified and segmented from the parafoveal character string. The CRM has successfully simulated a number of important eye movement characteristics associated with Chinese reading; however, in respect of the current experiments, it makes no quantitative predictions regarding how different types of parafoveal information will affect processing, nor how any such effects will change with development.

To date, no experiments have been conducted to investigate whether semantic parafoveal processing effects can be observed in children’s reading. The lack of such studies in the literature is perhaps not surprising given the inconsistency of such effects in investigations of adult processing (e.g., Liversedge et al., 2022; Schotter et al., 2012 for reviews). Also, it is more difficult to demonstrate effects in children’s reading relative to adults’ reading due to effects being smaller and eye movement data being more variable for children.

It might be reasonable, on a priori grounds, to suggest a hierarchy in relation to the depth to which parafoveal processing occurs in children. Orthographic parafoveal processing might be considered the shallowest level of parafoveal processing, phonological processing might be considered a somewhat deeper level of parafoveal processing, and finally, semantic parafoveal processing will likely be the deepest level of processing. Such a hierarchy may be plausible in reading of alphabetic language scripts, given that access to phonological and semantic form is likely gained via access to a stored orthographic form, and arguably, the phonological form of a word is representationally less rich than the semantic form of a word. If this characterization is reasonable, then we might also, tentatively, predict that the time course of development in efficacy of parafoveal processing with respect to each might adhere to this hierarchy relation. That is, in child readers, efficacy in orthographic parafoveal processing might develop first, followed by efficacy in parafoveal phonological and semantic processing. It may also be the case that parafoveal processing to a phonological level may precede parafoveal processing to a semantic level (should such processing be demonstrable at all). If these theoretical assumptions are accurate, then clearly, orthographic parafoveal processing effects should be apparent and more pronounced in younger children than phonological and semantic parafoveal effects. Similarly, potentially, phonological effects may also be present and more pronounced at a younger age than semantic parafoveal effects.

Only one study has currently published in a western journal that used eye movement recording methodology to investigate children’s parafoveal processing during Chinese reading. W. Zhou et al. (2018) employed an error disruption paradigm in which a group of third-grade (9.1 years) children and a group of adults read sentences that each contained a two-character target word. The target words were presented normally, or had their first character substituted for an alternative, unrelated character, or substituted by a character that was either phonologically or orthographically related to the identical character. In all three substitution conditions, the resulting two-character word was mis-spelled. Participants were required to read the sentences containing the mis-spellings and answer comprehension questions pertaining to their content. Given the prevalence of mis-spellings (75% of stimuli), it is not clear how their inclusion within sentences affected reading behavior. Nonetheless, Zhou et al.’s sought to determine readers’ parafoveal sensitivity to the different forms of mis-spelling to assess parafoveal processing in children and adults’ Chinese reading. They examined gaze duration for the two-character word that preceded the target word. If gaze durations for the pre-target word were inflated when the target was mis-spelled compared with when it was not, this would indicate a parafoveal-on-foveal sensitivity to the characteristics of the upcoming target. Furthermore, any differences between the mis-spelled conditions across participant groups might reflect differential sensitivity to parafoveal linguistic information. Zhou et al.’s results did show parafoveal-on-foveal effects with increased pre-target gaze durations for mis-spelled compared with correctly spelled words. Also, for the child readers, the pre-target word gaze durations were similar for the identical condition relative to when the target was mis-spelled but phonologically related, and both of these conditions yielded much reduced pre-target gaze durations compared with the unrelated condition. The orthographically related mis-spelled condition produced intermediate pre-target gaze durations. Two things are clear from this discussion. First, there is a paucity of experimental work to investigate children’s parafoveal processing during Chinese reading. Second, the single study that has been carried out strongly suggests that children aged approximately 9 years do engage in parafoveal processing during Chinese reading and that such processing is differentially sensitive to alternative forms of parafoveal linguistic information.

As discussed earlier, a more standard method of investigating parafoveal processing in reading is through the use of the boundary paradigm during natural reading. We carried out a series of boundary paradigm experiments to investigate children’s and adults’ parafoveal processing during natural Chinese reading, and how the nature of parafoveal processing developed across a range of ages. To do this, we tested five participant groups in each of our experiments: children in Grades 2, 3, 4, and 5 as well as a group of adults. In this way, we were able to chart developmental change in parafoveal processing with age. A further important aspect of our experiment concerns the nature of our parafoveal manipulations. As per our earlier discussion, we tentatively suggested a hierarchical relation between orthographic, phonological and semantic characteristics of words in relation to parafoveal processing in reading, and that there might be concordant processing development dependencies such that development of effective orthographic parafoveal processing might precede that of phonological and semantic processing, and in turn, potentially, development of effective phonological parafoveal processing might precede that of semantic processing. For this reason, in Experiment 1, we manipulated the orthographic characteristics of the preview relative to the target, in Experiment 2, the phonological relationship, and in Experiment 3, the semantic relationship. Broadly, we expected to demonstrate orthographic parafoveal sensitivity in younger children, phonological parafoveal sensitivity in children that were somewhat older, and any parafoveal sensitivity to semantic information that might be apparent would only occur for the adults and our oldest children. In all our experiments, the boundary directly preceded the first character of a one- or a two-character word^2^ and the pre-target word was always either one or two characters in length. We applied our parafoveal preview manipulation to the first character after the boundary and in all our analyses we computed eye movement measures for this single character region. We chose to apply our preview manipulation to the character after the boundary for three reasons. First, given that we were testing young children, and given that it is well established that young children have a limited perceptual span to the right of fixation (Yan et al., 2020), we wished to ensure that our preview manipulation fell within their perceptual span. Second, the application of the preview manipulation to the character immediately after the boundary ensured that preview effects were maximized for all our participant groups. In this way, the preview manipulation was as close to fixation prior to the boundary change as was possible. Third, since we manipulated preview in relation to only one character, we ensured that any effects we observed derived from change to a single linguistic unit. That is to say, there was no possibility of cross-character interference (in respect of orthography, phonology or semantic information). In all the experiments, we included an identical preview condition (the target character itself) and an unrelated condition (an unrelated character). We predicted that first pass reading time measures on the target would be shortest for the identical condition and longest for the unrelated condition. Critically, however, in Experiment 1, if children engaged in parafoveal orthographic processing, then orthographically related previews should convey a processing benefit relative to the unrelated preview condition. In Experiment 2, if children engaged in parafoveal phonological processing, then phonologically related previews should produce a processing benefit relative to the unrelated previews. And finally, in Experiment 3, if children engaged in semantic parafoveal processing, then semantically related previews should produce reduced reading times relative to the unrelated previews. And given our expectations for how parafoveal orthographic, phonological and semantic processing might develop with age, we expected to observe such reduced reading time effects (relative to the unrelated preview) at the earliest ages for the orthographically related stimuli in Experiment 1, and at somewhat later ages for the phonologically and semantically related stimuli in Experiments 2 and 3, with potentially delayed effects for semantic over phonological stimuli in Experiment 3 relative to Experiment 2.

## Experiment 1

In Experiment 1, we manipulated the orthography of previews to investigate whether children from Grade 2 to Grade 5 were able to effectively process parafoveal orthographic information.

### Method

#### Participants

One hundred twenty students from a primary school in Tianjin were recruited (30 Grade 2 participants, mean age = 7.93 years, standard deviation (*SD*) = 0.23; 30 Grade 3 participants, mean age = 8.96 years, *SD* = 0.23; 30 Grade 4 participants, mean age = 9.90 years, *SD* = 0.24; 30 Grade 5 participants, mean age = 11.02 years, *SD* = 0.25). Additionally, 30 undergraduate students (mean age = 19.30 years, *SD* = 1.57) from Tianjin Normal University took part. All of them had a normal or corrected to normal vision, and all were native readers of Chinese and all received a small gift for taking part in the experiment. All three experiments in this study were conducted according to the research ethical stipulations laid down by the British Psychological Society (Ethical Principles for Research with Human Participants. Ethical approval was not sought from Tianjin Normal University as no Ethics Committee existed in this institution at the time that the experiments were conducted. Briefly, the experiments were undertaken with informed consent, clear experiment instructions were provided, participants were free to withdraw at any time (without penalty), comprehensive debriefing was provided and all of the experimental data were anonymized, treated confidentially and remain securely stored.

#### Materials and design

The stimuli consisted of 60 target characters chosen from Pupils’ Dictionary of Homomorphic Characters. For each target character, three parafoveal previews were created. In the identical preview condition, the parafoveal previews were the same as target characters (mean frequency = 270.25 per million, *SD* = 682.24; mean number of strokes = 8.03, *SD* = 1.88). In the orthographically related preview condition, characters were orthographically similar to the target characters via a shared radical but were not phonologically or semantically related (mean frequency = 185.28 per million, *SD* = 464.99; mean number of strokes = 7.85, *SD* = 1.91). Finally, in the unrelated preview condition, characters were unrelated to the target (mean frequency = 162.71 per million, *SD* = 299.41; mean number of strokes = 8.15, *SD* = 1.60). All the orthographically related and unrelated preview characters were matched for frequency (*F*(2,118) = 1.18, *p* = .31) and stroke number (*F*(2,118) = 1.43, *p* = .24).

To confirm that related and unrelated preview characters were perceived as being visually similar or dissimilar to the targets we undertook a prescreening procedure. We asked 30 students from each of our participant groups to rate each preview/target character pair on a scale of 1 (*very visually different*) to 5 (*very visually similar*). The orthographically related preview and target character pair were rated as visually similar (mean similarity = 3.81, *SD* = 0.08), whilst the counterpart pairs were rated as visually dissimilar (orthographically related previews vs. unrelated previews, mean similarity = 1.00, *SD* = 0.00; targets vs. unrelated previews, mean similarity = 1.00, *SD* = 0.0). We conducted a 3 (similarity: targets vs. orthographically related previews; targets vs. unrelated previews; orthographically related previews vs. unrelated previews) × 5 (Grade: 2, 3, 4, 5, adult) ANOVA. Similarity was greater for orthographically related preview and target character pairs than for the other two pairs (*p*s < .001), and the difference in similarity for the other two pairs was not significant (*p* = .26). The main effect of grade (*F*(4,145) = 0.58, *p* = .68) and interaction (*F*(8, 290) = 0.58, *p* = .79) were not significant.

**Figure 1.**
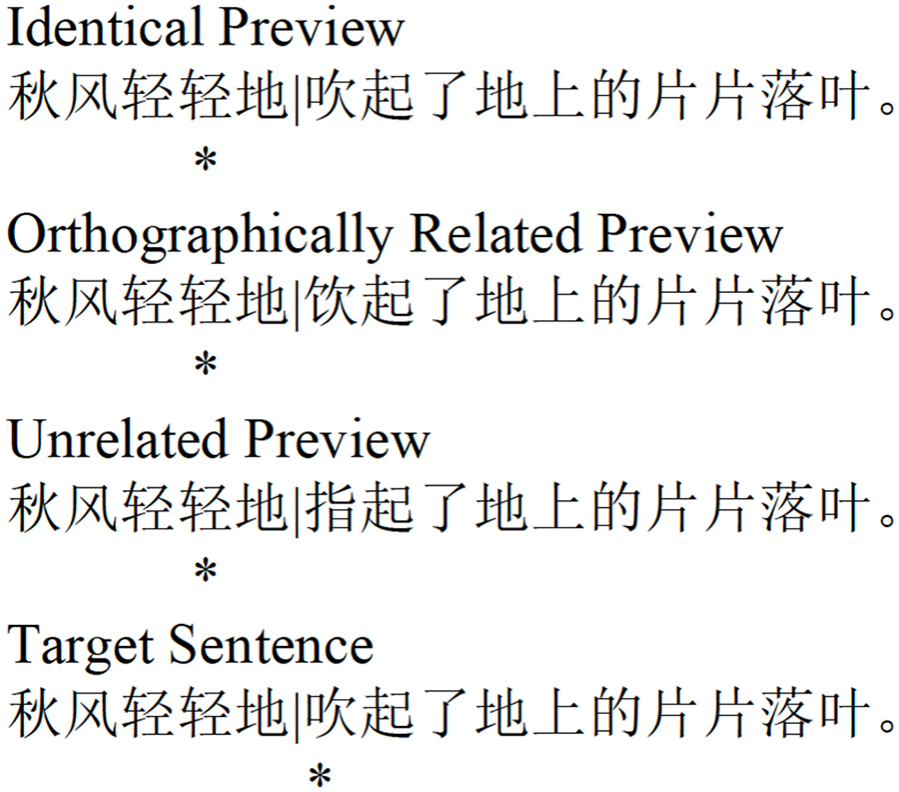
An example of an orthographic preview manipulation using the boundary paradigm. The target sentence is translated as “The Autumn wind gently blows the leaves on the ground.” The invisible boundary is positioned immediately prior to the target character 吹 (shown by a vertical line). Prior to the eyes crossing the boundary, the preview characters (吹, the identity preview; 饮 the orthographically related preview; 指 the orthographically unrelated preview) are presented at the position of the target character. When the point of fixation (indexed by asterisks) crosses the boundary during a saccade from left to right, the preview character is very rapidly replaced by the target character.

Sixty experimental sentence frames 14 to 18 characters long, written to be engaging and of a standard for second grade readers were generated (see Figure 1).

We also asked the participants in the pre-screen to rate the difficulty of the sentences on a scale of 1 (*very easy*) to 5 (*very difficult*). The sentences were quite easy to read (mean difficulty = 2.02, *SD* = 0.04) and there were no differences in difficulty across grades (*F*(4,149) = 1.48, *p* = .21). Moreover, another 30 participants in each grade assessed the naturalness of the sentences (5-point scale, 1 = *very natural*, 5 = *very unnatural*). Mean naturalness score was 2.01 (*SD* = 0.04), with no difference across grades (*F*(4,149) = 1.27, *p* = .29), indicating that the sentences were quite natural.

Finally, we tested a further 30 participants from each group in a cloze predictability study. The results showed that neither the orthographically related preview character nor the unrelated preview character was ever produced as a completion by any participant in any group. The identical preview character (e.g., target) was never produced by the children and extremely rarely by the adult participants (two participants each produced the target character for a single item). These results indicate that sentence contexts were not predictive of the target, and therefore, any orthographic preview benefit that might be obtained in the eye tracking experiment could not be attributed to target predictability. Finally, none of the participants from the rating studies participated in the eye tracking experiment.

The experimental design was a 5 (Grade: 2, 3, 4, 5, adult) × 3 (Target Character Preview: identical, orthographically related, unrelated) mixed design. We constructed three files, with each file containing 60 experimental sentences (20 sentences in each condition), 20 filler sentences without display changes and 6 practice sentences presented at the beginning of the experiment. The experimental conditions were rotated across files according to a Latin square, but sentences in each condition were presented randomly within a file. Each sentence was read only once by each participant. There were 20 comprehension questions (pertaining to 20 of our experimental stimuli) that participants were required to try to answer correctly with a yes/no response.

#### Apparatus

Eye movements were recorded using SR research EyeLink 2000 eye-tracker (sampling rate = 1,000 Hz). All stimuli were displayed at a distance of 67 cm from the participants on a Samsung SyncMaster 959NF 19-inch CRT monitor (refresh rate = 120 Hz, resolution = 1,024 × 768). Characters were presented in 23 Song font, which meant that each character occupied 1° of visual angle. Chin and forehead rests were used to minimize head movements.

#### Procedure

All participants were tested individually. They were seated comfortably, and then a 3-point single-line calibration and validation procedure were carried out. The six practice trials were then presented to participants, and then the formal experiment started. Participants were required to silently read and understand the sentences one by one. After the participant finished reading each sentence, they pressed a button on a gamepad to continue to the next trial, or to display a comprehension question. Participants answered the comprehension questions by making a yes/no response on the gamepad. Prior to each sentence, a drift-check point appeared on the left side of the screen. Fixations on the point initiated presentation of the next sentence, with its first character occupying the position of the drift-check point. In total, the experiment took about 30 min.

#### Power analysis

To formally demonstrate that we had sufficient power in our analyses, we considered the effects in a very related (though alphabetic) study by Tiffin-Richards and Schroeder, (2015). In this study, different types of preview effect (an orthographic preview effect and a phonological preview effect) were examined in a single experiment, and Tiffin-Richards et al. reported reliable preview effects with size (Cohen’s *d*) of between 0.60 and 0.72 for first-pass reading times (gaze duration, single fixation duration). Based on G-Power, the power of our current sample size (using 150 participants and 60 experimental stimuli in total) is estimated to be between 0.81 (*d* = 0.60) and 0.92 (*d* = 0.72). Using the pwrss package in R (Bulus, 2023), the power of our current sample size for the main effect of preview benefit is estimated to be between 0.89 and 0.97. Thus, our power analyses demonstrate that our experiments were sufficiently well powered to detect the preview effects we investigated.

All the data sets and R analysis code of the current study are available at https://osf.io/zj3un/.

### Results

Comprehension accuracy was high for all participants (range = 94.9%–95.7%, *M* = 95%, *SD* = 4.7%), indicating that participants read and understood the sentences. Consistent with previous eye movement experiments (e.g., Bai et al., 2008; Chace et al., 2005; White et al., 2005), all fixations less than 80 ms or greater than 1,200 ms were eliminated from the analyses. The following criteria were applied to filter trials: First, all trials were deleted if they received three or less fixations, and in this step, 0.49% of trials were excluded. Second, if a blink occurred during the display change or during a fixation on the target region, the trial was deleted. This removed 3.86% of the trials. Third, following previous literature (Cui et al., 2013, 2022), we removed trials where the display change occurred during a fixation rather than a saccade preceding the first fixation on the target character (7.04%). Finally, eye movement measures above or below 3 *SD*s from each participant’s mean were excluded (1.10%).

We undertook both global and local analyses of the data. Global analyses provide information about the general characteristics of reading across the entire sentence or text, whereas local analyses provide information about how a particular word or phrase was processed. Here, the global analyses are indicative of general changes in reading behavior between our participant groups (i.e., general changes with age), whilst the local analyses are reflective of how different target words were processed in relation to the different previews. Note also that effects observed in the local analyses will frequently be lost in the global analyses due to them being swamped by noise from the other words in the sentence. For the global analyses, we analyzed all the fixations made on the sentence. We computed four eye movement measures: total sentence reading times (the sum of all the fixations made on the sentence until the participant pressed the button to terminate the trial), number of fixations, average fixation duration, and mean rightward saccade extent. For the local analyses, we computed measures for the target character: first fixation duration (FFD, the duration of the first fixation on the target character), single fixation duration (SFD, the duration of the first fixation on the target character when it received only one fixation), gaze duration (GD, the sum of fixations on the target prior to a fixation on another character), go-past time (the sum of fixations from the first on the target until eyes fixated a character to its right), total time (the sum of all fixations on the target) and finally, skipping probability (percentage of times that readers did not fixate the target during first pass).

Eye movement data were analyzed by means of linear mixed models (LMM) using the lme4 package (version 4.3.1) in R (R Core Team, 2023). To increase the normality of the data, we natural log-transformed all the fixation duration measures before entering them into the analyses (for transparency, we report untransformed mean values). The number of fixations and skipping probability are discrete and binary variables respectively. Therefore, we used Poisson and binomial generalised linear mixed models (GLMMs) to analyze these measures respectively. For each dependent variable, a model was fit to the data with participants and items serving as crossed random effects. When conducting the global analyses, we specified Grade as a fixed factor. To minimize the number of comparisons, only when appropriate, we calculated contrasts comparing adjacent participant groups based on Grade (i.e., Grade 2 vs. 3; 3 vs. 4; 4 vs. 5; 5 vs. adults). For the local analyses, Grade and Target Character Preview were specified as fixed factors. The syntax for the global analyses was: (lmer (depvar ~ grade + (1|pp) + (1|stim), data = datafile)). The syntax for the local target character analyses was: (lmer (depvar ~ grade × preview + (1|pp) + (1|stim), data = datafile)).^3^ Effects were considered significant when |*t*| > 1.96. Finally, where appropriate, we calculated two contrasts for each dependent measure: identical preview condition versus orthographically related preview condition (the cost associated with a non-identical preview) and orthographically related preview condition versus unrelated preview condition (preview benefit). For brevity’s sake, fully specified interactions with respect to age, preview benefit and preview cost effects across Experiments 1 to 3 are provided in the online Supplemental Material.

#### Global analyses

As displayed in Tables 1 and 2, global eye movement measures showed a typical developmental pattern. There were trends towards shorter total reading times and fewer fixations with grade through to adults. The total reading time for second graders was numerically longer than for third graders (*b* = −0.18, *SE* = 0.09, *t* = −1.91, *p* = .06), and the number of fixations numerically greater for second than third graders (*b* = −0.13, *SE* = 0.08, *z* = −1.72, *p* = .08). Total reading time for third graders was significantly longer than for fourth graders (*b* = −0.29, *SE* = 0.09, *t* = −3.04, *p* < .01), with similar results for the number of fixations (*b* = −0.26, *SE* = 0.08, *z* = −3.42, *p* < .001). There was no significant difference between Grades 4 and 5 in both reading time and number of fixations (|*t*s| < 0.94, *p*s > .05), and the numerical difference was reduced relative to the magnitude of difference between younger grades. Nonetheless, the negative trend was maintained, and there is clearly developmental progression with respect to reduced reading times with age. Finally, the difference between adults and fifth graders in reading time and number of fixations was significant (|*t*s| > 2.38, *p*s < .05), and as we might expect, total times were shorter for adults than fifth graders.

**Table 1.** Means (and standard errors) of global eye movement measures.

Measures	Grade 2	Grade 3	Grade 4	Grade 5	Adult
Total sentence reading times (ms)	6,186 (90)	4,879 (49)	3,664 (40)	3,447 (42)	2,770 (35)
Number of fixations (*n*)	18.77 (0.24)	15.91 (0.15)	12.30 (0.12)	11.81 (0.13)	9.79 (0.10)
Average fixation duration (ms)	269 (1.02)	257 (1.10)	250 (1.21)	245 (0.97)	232 (0.93)
Rightward saccade extent (characters)	2.79 (0.03)	2.87 (0.02)	3.08 (0.03)	3.06 (0.03)	2.97 (0.02)

**Table 2.** Results of linear mixed effects on global eye movement measures.

Measures	β	*SE*	|*t*| or |*z*|	*p*
Total sentence reading times				
Intercept	8.18	0.03	258.22	<.001
Grade 2 vs. Grade 3	−.18	0.09	1.91	.06
Grade 3 vs. Grade 4	−.29	0.09	3.04	<.01
Grade 4 vs. Grade 5	−.09	0.09	0.93	.36
Grade 5 vs. Adult	−.23	0.09	2.39	.02
Number of fixations				
Intercept	2.54	0.03	95.06	<.001
Grade 2 vs. Grade 3	−.13	0.08	1.72	.08
Grade 3 vs. Grade 4	−.26	0.08	3.42	<.001
Grade 4 vs. Grade 5	−.06	0.08	0.81	.42
Grade 5 vs. Adult	−.20	0.08	2.56	.01
Average fixation duration				
Intercept	5.51	0.01	569.64	<.001
Grade 2 vs. Grade 3	−.05	0.03	1.64	.10
Grade 3 vs. Grade 4	−.03	0.03	1.01	.31
Grade 4 vs. Grade 5	−.01	0.03	0.43	.67
Grade 5 vs. Adult	−.06	0.03	1.85	.07
Rightward saccade extent				
Intercept	1.02	0.02	42.57	<.001
Grade 2 vs. Grade 3	.05	0.07	0.75	.46
Grade 3 vs. Grade 4	.06	0.07	0.85	.40
Grade 4 vs. Grade 5	.01	0.07	0.10	.92
Grade 5 vs. Adult	−.02	0.07	0.26	.80

Next, for the average fixation duration data, we see a similar numerical trend. Fixation durations decreased with grade through to adults, though the difference only approached significance between fifth-grade students and adults (*b* = −0.06, *SE* = 0.03, *t* = −1.85, *p* = .07). Finally, rightward saccade extent also showed a positive relation with grade, though again, these differences were not significant for the specific comparisons we made across grades through to adults (|*t*s| < 0.86, *p*s > .05). The lack of statistical significance for Grade differences in these measures is likely due to relatively small effect sizes and the relatively noisy data from the children. Furthermore, since we made formal comparisons only for adjacent grades to minimize comparisons in our analyses, the likelihood of group differences being significant was reduced (conducting non-adjacent group comparisons, e.g., Grade 2 vs. 5, most comparisons would have been statistically robust).

#### Local analyses

The mean values are provided in Table 3. Reading time measures in the identical preview condition were numerically shorter than those in the orthographically related preview condition across grades (i.e., there was a numerical preview cost); however, these differences were not consistently statistically robust.

**Table 3.** Orthographic preview effects: mean fixation durations (ms) and skipping probability, standard deviations appear in parentheses.

Grade	Preview type	First fixation duration	Single fixation duration	Gaze duration	Go-past time	Total time	Skipping probability
Grade 2	Identical	268 (131)	266 (136)	295 (166)	458 (459)	480 (322)	0.57 (0.50)
	Orthographically related	270 (124)	267 (120)	314 (178)	505 (478)	520 (359)	0.55 (0.50)
	Unrelated	296 (135)	289 (127)	338 (182)	595 (547)	509 (334)	0.57 (0.50)
	Orthographically related-Identical	2	1	19	47	40	−0.02
	Unrelated-Orthographically related	26*	22*	24*	90*	−11	0.02
Grade 3	Identical	246 (109)	247 (111)	261 (132)	440 (429)	391 (230)	0.57 (0.50)
	Orthographically related	268 (121)	265 (122)	293 (155)	470 (409)	431 (256)	0.56 (0.50)
	Unrelated	294 (135)	297 (138)	325 (160)	543 (421)	458 (256)	0.57 (0.50)
	Orthographically related-Identical	22^+^	18^+^	32*	30	40^+^	−0.01
	Unrelated-Orthographically related	26*	32*	32*	73**	27*	0.01
Grade 4	Identical	243 (102)	243 (99)	261 (134)	370 (329)	335 (202)	0.62 (0.49)
	Orthographically related	268 (122)	272 (124)	284 (129)	411 (335)	369 (223)	0.62 (0.49)
	Unrelated	287 (123)	291 (125)	307 (134)	453 (291)	374 (195)	0.57 (0.50)
	Orthographically related-Identical	25^+^	29^+^	23^+^	41^+^	34^+^	0.00
	Unrelated-Orthographically related	19*	19*	23*	42*	5	−0.05^+^
Grade 5	Identical	238 (86)	237 (85)	240 (90)	353 (290)	328 (183)	0.67 (0.47)
	Orthographically related	260 (104)	260 (107)	277 (124)	362 (258)	342 (195)	0.60 (0.49)
	Unrelated	277 (114)	278 (116)	296 (137)	410 (307)	382 (217)	0.52 (0.50)
	Orthographically related-Identical	22*	23*	37**	9	14	−0.07*
	Unrelated-Orthographically related	17*	18*	19^+^	48*	40**	−0.08**
Adult	Identical	226 (76)	227 (76)	230 (79)	331 (252)	282 (153)	0.64 (0.48)
	Orthographically related	244 (88)	244 (88)	254 (108)	336 (260)	298 (157)	0.59 (0.49)
	Unrelated	277 (123)	278 (123)	288 (129)	428 (330)	324 (179)	0.64 (0.48)
	Orthographically related-Identical	18^+^	17^+^	24*	5	16	−0.05^+^
	Unrelated-Orthographically related	33*	34*	34**	92***	26	0.05

*Note*. The Identical-Orthographically related differences represent the cost to processing of having an orthographically related preview relative to an identical preview. The Unrelated-Orthographically related differences represent the orthographic preview benefit associated with having an orthographically related preview relative to an unrelated preview.

+ *p* < .10. *p < .05. **p < .01. ***p < .001.

**Figure 2.**
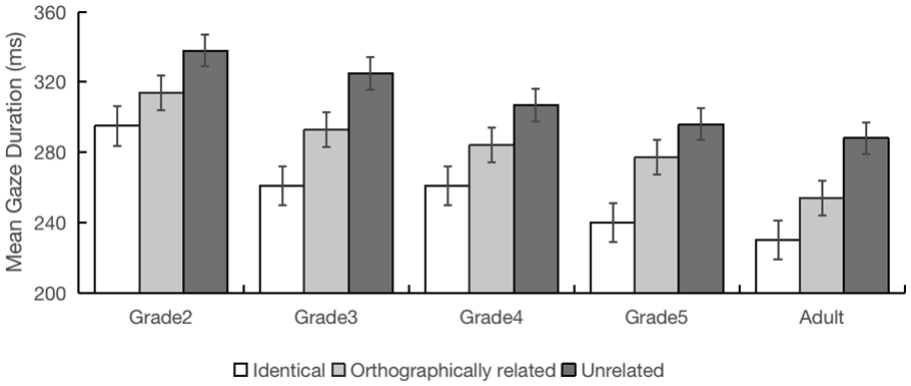
The orthographic preview effects in gaze duration for the five participant groups (error bars represent standard errors of the mean).

Further, all grades showed orthographic preview benefit effects across FFD, SFD, GD, and go-past time measures (|*t*s| > 2.03, *p*s < .05), though the difference for fifth graders only approached significance in GD (*b* = 0.07, *SE* = 0.04, *t* = 1.91, *p* = .06) (see Figure 2). It is noteworthy that the preview cost and preview benefit effects for the skipping data were not significant, other than for the fifth-grade participants. Also, skipping probability was high (between 0.52 and 0.67). The lack of robust effects for this measure, alongside the high skipping rates, very likely occurred because the target was the first character of a two-character word, and many fixations would land on the second character of this word. This makes it difficult to meaningfully interpret the skipping data. Overall, these results demonstrate that children across all grades (as well as adults) processed orthographically related previews effectively prior to their direct fixation.^4^

### Discussion

The results of Experiment 1 showed a typical developmental trajectory: fixation durations and counts reduced, while saccade extent increased with grade. These results are consistent with the results of previous studies (e.g., Pagán et al., 2016; Tiffin-Richards & Schroeder, 2015) and demonstrate that, generally, as readers become more proficient with increasing grade, they make fewer and shorter fixations and their ability to effectively process stimuli in the parafovea increases.

The main question we addressed in Experiment 1 was whether even the youngest children in our study (Grade 2) could effectively process orthographically related parafoveal previews to produce preview benefit effects when the target was directly fixated. We anticipated that of the three parafoveal manipulations we employed across our experiments, this manipulation was the most likely to produce effects for all grades. Indeed, the results showed significant differences between orthographically related and unrelated preview conditions across first-pass reading time measures (FFD, SFD, and GD) and go-past time for all grades, indicating that all participant groups, including the youngest second graders, could obtain useful orthographic information from the upcoming character. This finding shows that readers extracted and processed visual characteristics of the target character prior to its fixation. Note also that the preview benefit effect we observed for Chinese (between 19 and 34 ms for gaze duration) was comparable to orthographic preview benefit effects reported in alphabetic languages (e.g., Pagán et al., 2016).

It is documented that readers not only process orthographic information from upcoming text but also phonological information, and arguably, semantic information (Blythe et al., 2018; Pan et al., 2016; Tiffin-Richards & Schroeder, 2015; Tsai et al., 2012; Yan et al., 2009; Yang et al., 2012; W. Zhou et al., 2018). As discussed earlier, phonological and semantic information associated with words are sources of linguistic (not visual) information and might, therefore, be considered higher order forms of information. If this is the case, then the developmental trajectory of these types of parafoveal processing may lag behind that of orthographic parafoveal processing, meaning that younger readers may not exhibit a sensitivity to phonological and semantic preview information relative to older children. Experiments 2 and 3 investigated this question.

## Experiment 2

We investigated whether children from Grade 2 to Grade 5, as well as adults, were able to effectively process phonological preview information.

### Method

#### Participants

One hundred twenty-six primary school students from Tianjin were tested (30 Grade 2 participants, mean age = 7.96 years, *SD* = 0.26; 33 Grade 3 participants, mean age = 8.95 years, *SD* = 0.23; 30 Grade 4 participants, mean age = 9.92 years, *SD* = 0.24; 33 Grade 5 participants, mean age = 10.95 years, *SD* = 0.20). Additionally, 30 undergraduate students (mean age = 19.57 years, *SD* = 1.31) from Tianjin Normal University took part. None of these participants took part in Experiment 1. All participants had normal or corrected-to-normal vision, and all were native readers of Chinese. All of the participants received a small gift for taking part.

#### Materials and design

The stimuli consisted of 60 target characters selected from the Pupils’ Dictionary of Homophonic Characters. For each target character, three parafoveal previews were generated. In the identical preview condition, the parafoveal previews were the same as the target characters (mean frequency = 221.58 per million, *SD* = 766.88; mean number of strokes = 7.77, *SD* = 2.24). In the phonologically related preview condition, preview characters had the same phonology as the target characters, but were not orthographically or semantically related (mean frequency = 396.78 per million, *SD* = 882.94; mean number of strokes = 7.28, *SD* = 3.03). Finally, in the unrelated preview condition, characters were unrelated to the target characters (mean frequency = 353.84 per million, *SD* = 796.34; mean number of strokes = 7.42, *SD* = 2.21). An ANOVA showed that all the phonologically related and unrelated preview characters were matched with targets for frequency (*F*(2,118) = 0.76, *p* = .47) and stroke number (*F*(2,118) = 0.88, *p* = .42).

To confirm that preview character pairs were comparably orthographically dissimilar to the target, we undertook a pre-screen. We asked 30 students from each of our participant groups to rate each preview character pair (relative to the target) on a scale of 1 (*very visually different*) to 5 (*very visually similar*). The ratings indicated that each preview pair was visually different (phonologically related previews vs. targets, mean similarity = 1.00, *SD* = 0.00; unrelated previews vs. targets, mean similarity = 1.00, *SD* = 0.01; phonologically related previews vs. unrelated previews, mean similarity = 1.00, *SD* = 0.00). Also, these data were analyzed using a 3 (similarity: target vs. phonologically related previews; target vs. unrelated previews, phonologically related previews vs. phonologically unrelated previews) × 5 (Grade: 2, 3, 4, 5, adult) ANOVA. The results showed no main effect of similarity (*F*(2,290) = 0.33, *p* = .72), grade (*F*(4,145) = 0.14, *p* = .97) nor an interaction (*F*(8,290) = 0.53, *p* = .83), providing no evidence for differences in similarity between conditions and across grades. None of the pre-screen participants took part in the eye tracking test.

Sixty experimental sentence frames written to be engaging and of a standard for second graders were generated. See Example (1) with the identical preview character (target), phonologically related preview character, and unrelated preview character in bold.

(1) 明亮温暖的**阳/洋/拉**光轻轻地洒落在草原上。

(Translation: The bright and warm **sun/ocean/drawing** gently shines on the grassland.)

All the sentences were between 14 and 18 characters. We also asked the visual similarity pre-screen participants to rate the difficulty of the sentences on a scale of 1 (*very easy*) to 5 (*very difficult*) and showed that the sentences were quite easy to read (mean difficulty = 2.02, *SD* = 0.04) and that there were no differences in sentence difficulty across grade (*F*(4,149) = 0.82, *p* = .51). Moreover, another 30 participants in each grade were required to assess the naturalness of the sentences (5-point scale, 1 = *very natural*; 5 = *very unnatural*). The mean naturalness score was 1.99 (*SD* = 0.03), with no difference in naturalness across grades (*F*(4,149) = 1.19, *p* = .32). The stimuli were, therefore, similarly easy to read and natural.

A cloze predictability study using another 30 students examined how often readers predicted the critical words from prior sentence context. The phonologically related preview character, or unrelated preview character were never produced as a completion by any participant in any group. The identical preview character (i.e., target) was never produced by the children and only by four adult participants for a different single item each. The sentence contexts were not predictive of the target. Again, none of the participants from the rating studies participated in the eye tracking test.

The experimental design was a 5 (Grade: 2, 3, 4, 5, adult) × 3 (Target Character Preview: identical, phonologically related, unrelated) mixed design. We constructed three files with each file containing 60 experimental sentences (20 sentences in each condition), 20 filler sentences without display changes, and 6 practice sentences presented at the beginning of the experiment. The experimental conditions were rotated across files according to a Latin square, but sentences in each condition were presented randomly within a file. Each sentence was read only once by each participant. There were 20 comprehension questions (pertaining to 20 of our experimental stimuli) that participants were required to answer correctly with a yes/no response.

#### Apparatus, procedure and power analysis

Identical to Experiment 1.

### Results

Comprehension accuracy was high (range = 91.5%–96.5%, *M* = 93.1%, *SD* = 4.5%), indicating that all participants understood the sentences. The data deletion criteria were identical to Experiment 1. Trials were deleted if the fixation number was less than three (0.83% of trials), when a blink occurred during the display change or during a fixation on the target region (3.2%), or when the display change occurred during a fixation rather than a saccade preceding the first fixation on the target character (7.51%). Eye movement measures above or below 3 *SD*s from each participant’s mean were also excluded (1.3% of trials).

We used the same eye movement measures and LMMs as in Experiment 1.

#### Global analyses

As displayed in Tables 4 and 5, again, the global measures showed a typical developmental pattern. In total reading time and number of fixations, there were trends towards shorter times and fewer fixations with grade through to adults, though the numerical difference was not significant between Grade 5 and adults for total reading time (*b* = −0.16, *SE* = 0.09, *t* = −1.77, *p* = .08) and only significant between Grade 5 and adults in number of fixations (*b* = −0.17, *SE* = 0.07, *z* = −2.29, *p* = .02). Similar numerical patterns occurred for average fixation duration and rightward saccade extent, though these were not significant (|*t*s| < 1.02, *p*s > .05).

**Table 4.** Means (and standard errors) of global eye movement measures.

Measures	Grade 2	Grade 3	Grade 4	Grade 5	Adult
Total sentence reading times (ms)	4,940 (61)	4,338 (48)	4,286 (62)	3,619 (39)	3,036 (28)
Number of fixations (*n*)	15.91 (0.19)	13.96 (0.14)	13.68 (0.18)	12.19 (0.13)	10.17 (0.09)
Average fixation duration (ms)	273 (1.10)	266 (1.12)	261 (1.12)	259 (1.20)	259 (1.12)
Rightward saccade extent (characters)	2.54 (0.03)	2.71 (0.03)	2.81 (0.02)	2.68 (0.02)	2.51 (0.02)

**Table 5.** Results of linear mixed effects on global eye movement measures.

Measures	β	*SE*	|*t*| or |*z*|	*p*
Total sentence reading times				
Intercept	8.17	0.03	268.50	<.001
Grade 2 vs. Grade 3	−.12	0.09	1.33	.19
Grade 3 vs. Grade 4	−.08	0.09	0.85	.40
Grade 4 vs. Grade 5	−.10	0.09	1.12	.26
Grade 5 vs. Adult	−.16	0.09	1.77	.08
Number of fixations				
Intercept	2.52	0.03	91.83	<.001
Grade 2 vs. Grade 3	−.11	0.07	1.54	.12
Grade 3 vs. Grade 4	−.07	0.07	0.92	.36
Grade 4 vs. Grade 5	−.08	0.07	1.04	.30
Grade 5 vs. Adult	−.17	0.07	2.29	.02
Average fixation duration				
Intercept	5.56	0.01	562.82	<.001
Grade 2 vs. Grade 3	−.03	0.03	1.01	.31
Grade 3 vs. Grade 4	−.02	0.03	0.62	.54
Grade 4 vs. Grade 5	−.01	0.03	0.26	.80
Grade 5 vs. Adult	−.00	0.03	0.01	.99
Rightward saccade extent				
Intercept	.91	0.02	40.50	<.001
Grade 2 vs. Grade 3	.06	0.07	0.94	.35
Grade 3 vs. Grade 4	.06	0.07	0.92	.36
Grade 4 vs. Grade 5	−.05	0.07	0.71	.48
Grade 5 vs. Adult	−.06	0.07	0.82	.42

#### Local analyses

The means and standard deviations of the local measures are shown in Table 6.

**Table 6.** Phonological preview effects: mean fixation durations (ms) and skipping probability, standard deviations appear in parentheses.

Grade	Preview type	First fixation duration	Single fixation duration	Gaze duration	Go-past time	Total time	Skipping probability
Grade 2	Identical	274 (118)	273 (116)	297 (140)	457 (410)	422 (266)	0.51 (0.50)
	Phonologically related	321 (154)	319 (152)	359 (194)	585 (486)	495 (290)	0.51 (0.50)
	Unrelated	314 (168)	311 (163)	345 (195)	558 (414)	483 (279)	0.45 (0.50)
	Phonologically related-Identical	47***	46***	62***	128***	73***	0
	Unrelated-Phonologically related	−7	−8	−14	−27	−12	−0.06*
Grade 3	Identical	260 (101)	261 (102)	277 (116)	395 (316)	373 (210)	0.54 (0.50)
	Phonologically related	310 (127)	313 (131)	344 (151)	502 (333)	425 (227)	0.49 (0.50)
	Unrelated	302 (133)	298 (139)	335 (175)	551 (454)	446 (270)	0.47 (0.50)
	Phonologically related-Identical	50***	52***	67***	107***	52***	−0.05
	Unrelated-Phonologically related	−8	−15	−9	49	21	−0.02
Grade 4	Identical	258 (113)	254 (108)	275 (140)	408 (366)	389 (259)	0.58 (0.49)
	Phonologically related	288 (130)	288 (136)	320 (161)	480 (376)	413 (278)	0.59 (0.49)
	Unrelated	307 (121)	306 (114)	341 (157)	515 (356)	443 (295)	0.55 (0.50)
	Phonologically related-Identical	30*	34**	45**	72**	24	0.01
	Unrelated-Phonologically related	19*	18*	21*	35^+^	30*	−0.04
Grade 5	Identical	238 (98)	239 (100)	251 (111)	375 (350)	327 (186)	0.59 (0.49)
	Phonologically related	265 (106)	261 (104)	279 (122)	412 (309)	345 (187)	0.55 (0.50)
	Unrelated	292 (119)	288 (118)	326 (160)	461 (328)	393 (212)	0.53 (0.50)
	Phonologically related-Identical	27**	22**	28**	37*	18*	−0.04
	Unrelated-Phonologically related	27**	27**	47***	49*	48**	−0.02
Adult	Identical	232 (75)	235 (73)	238 (76)	306 (210)	280 (136)	0.60 (0.49)
	Phonologically related	268 (96)	266 (95)	276 (108)	360 (220)	324 (160)	0.56 (0.50)
	Unrelated	286 (107)	284 (103)	299 (120)	368 (211)	345 (196)	0.47 (0.50)
	Phonologically related-Identical	36***	31**	38***	54***	44***	−0.04
	Unrelated-Phonologically related	18*	18*	23**	8	21	−0.09**

*Note*. The Identical-Phonologically related differences represent the cost to processing of having a phonologically related preview relative to an identical preview. The Unrelated-Phonologically related differences represent the phonological preview benefit associated with having a phonologically related preview relative to an unrelated preview.

+ *p* < .10. *p < .05. **p < .01. ***p < .001.

The reading time measures for the identical condition were significantly shorter than for the phonologically related condition across grades (|*t*s| > 1.98, *p*s < .05), other than for the fourth graders on total reading time (*b* = −0.04, *SE* = 0.04, *t* = −0.99, *p* = .32). In the skipping probability, there were no reliable effects between the identical and related conditions across all the grades (|*t*s| < 1.64, *p*s > .05).

**Figure 3.**
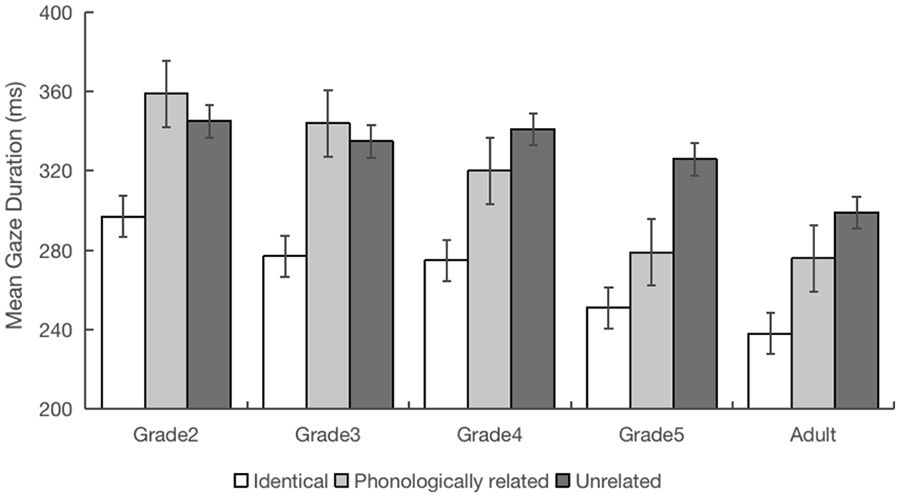
The phonological preview effects in gaze duration for the five participant groups (error bars represent standard errors of the mean).

There were no significant phonological preview benefit effects in the second and third grade participants for any of the reading time measures (|*t*s| < 1.74, *p*s > .05). However, we did observe a significant phonological preview benefit in the fourth, fifth and adult participants across the measures of FFD, SFD, GD (|*t*s| > 2.00, *p*s < .05) (see Figure 3). For fourth graders, there was a numerical (non-significant) preview benefit effect on go-past time (*b* = 0.11, *SE* = 0.06, *t* = 1.91, *p* = .06), and a significant effect for total reading time (*b* = 0.10, *SE* = 0.04, *t* = 2.55, *p* = .01). The preview benefit effects were statistically robust on the measures of go-past time and total reading time for fifth graders (|*t*s| > 2.40, *p*s < .05); however, these effects were not significant for go-past and total reading times in adult readers (|*t*s| < 1.58, *p*s > .05). Whilst skipping probabilities were significantly larger in the related than unrelated conditions for second grade and adult readers (|*t*s| > 2.20, *p*s < .05), there was no such difference for the other grades (|*t*s| < 1.28, *p*s > .05).

Overall, the results provide little evidence that participants from Grades 2 and 3 obtained phonological preview benefit from the upcoming character, but that participants in Grades 4 and 5, as well as adults did. Thus, in contrast to the results from Experiment 1, here in Experiment 2, it appears that effective phonological processing of upcoming Chinese characters was developmentally delayed relative to effective orthographic processing, with parafoveal phonological processing developing and maintaining from the fourth grade in contrast to parafoveal orthographic processing which occurred and maintained from the second grade onwards.

### Discussion

Experiment 2 explored whether students from Grade 2 to 5 obtained phonological preview benefit. Our global analyses showed typical developmental eye movement patterns consistent with the results from Experiment 1. The local analyses showed that phonological preview benefit appeared in fourth grade and older readers, but not in the participants younger than this. There appears to be a step change between the Grade 3 and Grade 4 readers in the degree to which phonological parafoveal processing occurs during natural Chinese reading. Thus, development of phonological processing of upcoming text appears delayed relative to counterpart orthographic processing.

At first sight, our findings may be considered somewhat inconsistent with the findings of W. Zhou et al. (2018) who demonstrated that third grade participants exhibited phonological parafoveal-on-foveal effects on pre-target words in sentence reading. As with the present experiment, Zhou et al. also manipulated the first character of a two character word. It is likely that differences in the stimuli between the studies explains these inconsistent findings. In the present experiment 93% of our target characters were compound characters, that is, characters formed from two or more independent radicals, and only 7% of our targets were integrated characters that were not comprised of separable radicals. Furthermore, of the compound characters that formed the targets in the present experiment, over 70% were comprised of radicals that did not convey phonetic information. In contrast, in Zhou et al.’s study, 89% of the target characters were high frequency and orthographically simple integrated characters that conveyed phonetic information via their unified entirety (no separable radicals). It seems likely, therefore, that integrated characters are more effective in conveying phonetic information (at least in relation to parafoveal processing) than are compound characters used in Experiment 2 (due to differences in the nature of the characters, their frequency or their visual complexity). A further important difference between the studies concerns the task. In Zhou et al.’s experiment, participants were required to read sentences that included characters that did not make sense within the context, and they were forewarned that the sentences were of this type. This task is quite different to natural reading.

Experiments 1 and 2 examined the development of parafoveal orthographic and phonological processing. In our third experiment, we explored the development of parafoveal semantic processing.

## Experiment 3

In Experiment 3 we investigated whether children from Grade 2 to Grade 5 showed semantic preview benefit effects during natural Chinese reading.

### Method

#### Participants

A total of 120 primary school students from Tianjin were tested (30 Grade 2 participants, mean age = 7.91 years, *SD* = 0.23; 30 Grade 3 participants, mean age = 8.95 years, *SD* = 0.25; 30 Grade 4 participants, mean age = 9.91 years, *SD* = 0.24; 30 Grade 5 participants, mean age = 10.96 years, *SD* = 0.20). Additionally, 30 undergraduate students from Tianjin Normal University took part (mean age = 19.87 years, *SD* = 1.24). There was some overlap in the participants that took part in Experiments 2 and 3 (Grade 2, 66.7% overlap, Grade 3, 73.3% overlap, Grade 4, 53.3% overlap, Grade 5, 60% overlap, adults, 0% overlap). The overlap occurred due to participant availability in relation to the testing schedule that was required. All the participants had a normal or corrected to normal vision, were native readers of Chinese and received a gift for participating.

#### Materials and design

The stimuli consisted of 60 target characters chosen from Pupils’ Dictionary of Synonym. For each target, three parafoveal previews were generated. In the identical preview condition, the previews were the same as the target characters (mean frequency = 352.84 per million, *SD* = 737.69; mean number of strokes = 7.70, *SD* = 2.55). In the semantically related preview condition, preview characters were semantically related to the target characters, but were not orthographically or phonologically related (mean frequency = 405.95 per million, *SD* = 834.38; mean number of strokes = 7.78, *SD* = 3.02). Finally, in the unrelated preview condition, characters were unrelated to the target characters (mean frequency = 334.74 per million, *SD* = 941.01; mean number of strokes = 7.97, *SD* = 2.27). ANOVA results showed all the semantically related and unrelated preview characters were matched with targets for frequency (*F*(2,118) = 0.12, *p* = .89)^5^ and constituent stroke number (*F*(2,118) = 0.23, *p* = .80).

Again, we undertook a pre-screen to evaluate the visual similarity of each preview character pair in relation to the target. Thirty students from each participant group rated each preview character pair for visual similarity relative to the target. The ratings indicated that each pair was visually different (semantically related previews vs. targets, mean similarity = 1.00, *SD* = 0.01; unrelated previews vs. targets, mean similarity = 1.00, *SD* = 0.01; semantically related previews vs. unrelated previews, mean similarity = 1.00, *SD* = 0.01), and a 3 (similarity: targets vs. semantically related previews; targets vs. unrelated previews, semantically related previews vs. unrelated previews) × 5 (Grade: 2, 3, 4, 5, adult) ANOVA showed no effect of similarity (*F*(2,290) = 2.25, *p* = .11), grade (*F*(4,145) = 0.15, *p* = .97), nor interaction (*F*(8,290) = 0.57, *p* = .80). The pre-screen stimuli were matched for visual similarity.

In addition, we evaluated the semantic similarity of our previews to the target. Another 30 students from each of our participant groups rated each preview character pair on a scale of 1 (*very semantically unrelated*) to 5 (*very semantically related*). Unsurprisingly, semantically related preview and target character pairs were related (mean semantic relatedness = 4.31, *SD* = 0.08), unrelated and target pairs were unrelated (targets vs. unrelated previews, mean semantic relatedness = 1.00, *SD* = 0.00). A 2 (semantic relatedness: targets vs. semantically related previews; targets vs. unrelated previews) × 5 (Grade: 2, 3, 4, 5, adult) ANOVA showed that the related preview and target character pairs were much more related than was the case for the unrelated pairs (*F*(1,145) = 256,480.56, *p* < .001). The main effect of grade (*F*(4,145) = 0.76, *p* = .56) and interaction (*F*(4,145) = 0.71, *p* = .59) were not significant. None of the pre-screen participants took part in the eye tracking test.

Sixty experimental sentence frames written to be engaging and of a standard for second graders were generated. See Example (2) with the identical preview character (target), semantically related preview character, and unrelated preview character in bold.

(2) 冰箱里拿出来的**冷/寒/团**水冒着白色的烟雾。

(Translation: The **cold/freezing/round** water from the refrigerator was white and misty.)

All the sentences were between 14 to 18 characters. We also asked the visual similarity pre-screen participants to rate the difficulty of the sentences on a scale of 1 (*very easy*) to 5 (*very difficult*). The sentences were quite easy to read (mean difficulty = 2.03, *SD* = 0.04) and that there were no significant differences in sentence difficulty across grade (*F*(4,149) = 1.94, *p* = .11). Moreover, another 30 assess the stimuli for naturalness. The mean naturalness score was 2.01 (*SD* = 0.04), with no difference in the naturalness across grades (*F*(4,149) = 0.71, *p* = .59). Stimuli were matched on visual similarity and naturalness.

A cloze predictability rating study using another 30 students was conducted to assess target predictability. The semantically related preview character, or unrelated preview character, was never produced as a completion by any participant in any group. The identical preview character (i.e., target) was never produced by the children and only six adult participants produced the target character for a different single item each. These results indicate that sentence contexts were not predictive of the target. Again, none of the participants from the rating studies participated in the eye tracking test.

The experimental design was a 5 (Grade: 2, 3, 4, 5, adult) × 3 (Target Character Preview: identical, semantically related, unrelated) mixed design. We constructed three files with each file containing 60 experimental sentences (20 sentences in each condition), 20 filler sentences without display changes, and 6 practice sentences presented at the beginning of the experiment. The experimental conditions were rotated across files according to a Latin square, but sentences in each condition were presented randomly within a file. Each sentence was read only once by each participant. There were 20 comprehension questions answered with a yes/no response.

#### Apparatus, procedures and power analysis

Identical to Experiment 1.

### Results

Comprehension accuracy was high for all participants (range = 91.5%–95.7%, *M* = 94.4%, *SD* = 4.8%). The approach to data deletion and analysis was the same as Experiment 1. Trials were deleted with less than three fixations (0.52%), if a blink occurred during the display change or a fixation on the target (2.62%), if the display change occurred during a fixation (7.11%), and measures above or below 3 *SD*s the participant mean were excluded (1.19%).

We used the same eye movement measures and LMMs as in Experiment 1.

#### Global analyses

As shown in Tables 7 and 8, we obtained the standard developmental pattern as in Experiments 1 and 2. In total reading time and number of fixations, there were linear trends towards shorter total reading times and fewer fixations with grade through to adults. The total reading time for the second graders was numerically longer than for third graders (*b* = −0.16, *SE* = 0.09, *t* = −1.68, *p* = .09), and the number of fixations numerically greater for second than third graders (*b* = −0.14, *SE* = 0.08, *z* = −1.83, *p* = .07). Total reading time for third graders was significantly longer than for fourth graders (*b* = −0.24, *SE* = 0.09, *t* = −2.59, *p* = .01), with similar results for the number of fixations (*b* = −0.21, *SE* = 0.08, *z* = −2.74, *p* = .01). There was no significant difference between Grades 4 and 5 in both total reading time and number of fixations (*|t*s*|* < 1.49, *p*s > .05), and the numerical difference was reduced relative to the magnitude of difference between younger grades. Nonetheless, the negative trend maintained and there was clearly developmental progression with respect to reduced reading times with age. Finally, the difference between adults and fifth graders in reading time and number of fixations was significant (*|t*s*|* > 3.07, *p*s < .01), and as we might expect, total times were shorter for adults than fifth graders.

**Table 7.** Means (and standard errors) of global eye movement measures.

Measures	Grade 2	Grade 3	Grade 4	Grade 5	Adult
Total sentence reading times (ms)	5,350 (63)	4,596 (63)	3,516 (39)	4,023 (47)	3,014 (33)
Number of fixations (*n*)	16.39 (0.18)	14.30 (0.16)	11.63 (0.11)	13.28 (0.15)	10.49 (0.11)
Average fixation duration (ms)	281 (1.22)	271 (1.21)	261 (1.13)	263 (1.21)	247 (1.08)
Rightward saccade extent (characters)	2.64 (0.03)	2.55 (0.02)	2.96 (0.03)	2.59 (0.02)	2.58 (0.02)

**Table 8.** Results of linear mixed effects on global eye movement measures.

Measures	β	*SE*	|*t*| or |*z*|	*p*
Total sentence reading times				
Intercept	8.18	0.03	254.25	<.001
Grade 2 vs. Grade 3	−.16	0.09	1.68	.09
Grade 3 vs. Grade 4	−.24	0.09	2.59	.01
Grade 4 vs. Grade 5	.13	0.09	1.38	.17
Grade 5 vs. Adult	−.29	0.09	3.07	<.01
Number of fixations				
Intercept	2.52	0.03	90.58	<.001
Grade 2 vs. Grade 3	−.14	0.08	1.83	.07
Grade 3 vs. Grade 4	−.21	0.08	2.74	.01
Grade 4 vs. Grade 5	.11	0.08	1.48	.14
Grade 5 vs. Adult	−.24	0.08	3.07	<.01
Average fixation duration				
Intercept	5.56	0.01	538.42	<.001
Grade 2 vs. Grade 3	−.04	0.03	1.19	.24
Grade 3 vs. Grade 4	−.04	0.03	1.11	.27
Grade 4 vs. Grade 5	.00	0.03	0.14	.89
Grade 5 vs. Adult	−.06	0.03	1.93	.06
Rightward saccade extent				
Intercept	.92	0.02	40.62	<.001
Grade 2 vs. Grade 3	−.01	0.07	0.10	.92
Grade 3 vs. Grade 4	.14	0.07	2.04	.04
Grade 4 vs. Grade 5	−.12	0.07	1.80	.07
Grade 5 vs. Adult	.01	0.07	0.13	.90

For the average fixation duration data, there was a very similar numerical trend such that fixation durations decreased with grade through to adults, though the difference only approached significance between fifth grade students and adults (*b* = −0.06, *SE* = 0.03, *t* = −1.93, *p* = .06). Finally, the rightward saccade extent results did not show clear numerical trends, though there were significant differences between third grade and fourth grade students (*b* = 0.14, *SE* = 0.07, *t* = 2.04, *p* = .04), and an effect that approached significance between fourth and fifth graders (*b* = −0.12, *SE* = 0.07, *t* = −1.80, *p* = .07).

#### Local analyses

The mean values are provided in Table 9.

**Table 9.** Semantic preview effects: mean fixation durations (ms) and skipping probability, standard deviations appear in parentheses.

Grade	Preview type	First fixation duration	Single fixation duration	Gaze duration	Go-past time	Total time	Skipping probability
Grade 2	Identical	285 (138)	277 (137)	322 (189)	521 (512)	502 (344)	0.52 (0.50)
	Semantically related	321 (159)	319 (164)	375 (220)	615 (559)	566 (378)	0.49 (0.50)
	Unrelated	316 (164)	310 (163)	367 (214)	645 (595)	550 (355)	0.49 (0.50)
	Semantically related-Identical	36**	42**	53***	94**	64*	−0.03
	Unrelated-Semantically related	−5	−9	−8	30	−16	0.00
Grade 3	Identical	276 (114)	271 (106)	298 (148)	465 (417)	454 (330)	0.51 (0.50)
	Semantically related	294 (135)	286 (128)	316 (166)	512 (430)	481 (324)	0.48 (0.50)
	Unrelated	305 (131)	302 (133)	342 (164)	565 (469)	487 (305)	0.47 (0.50)
	Semantically related-Identical	18	15	18	47	27*	−0.03
	Unrelated-Semantically related	11	16	26*	53	6	−0.01
Grade 4	Identical	260 (101)	261 (103)	277 (134)	381 (337)	360 (215)	0.61 (0.49)
	Semantically related	289 (130)	285 (127)	304 (161)	427 (352)	422 (247)	0.57 (0.49)
	Unrelated	306 (149)	304 (155)	347 (209)	483 (362)	433 (264)	0.55 (0.50)
	Semantically related-Identical	29*	24*	27^+^	46^+^	62***	−0.04
	Unrelated-Semantically related	17	19	43*	56*	11	−0.02
Grade 5	Identical	252 (108)	253 (108)	273 (130)	390 (309)	384 (237)	0.50 (0.50)
	Semantically related	282 (125)	282 (134)	305 (159)	438 (334)	452 (265)	0.52 (0.50)
	Unrelated	311 (135)	318 (141)	335 (150)	509 (355)	433 (242)	0.44 (0.50)
	Semantically related-Identical	30**	29*	32*	48^+^	68***	0.02
	Unrelated-Semantically related	29**	36**	30**	71**	−19	−0.08**
Adult	Identical	250 (91)	250 (90)	258 (103)	327 (233)	306 (167)	0.50 (0.50)
	Semantically related	276 (126)	267 (113)	292 (147)	368 (260)	368 (241)	0.55 (0.50)
	Unrelated	299 (128)	300 (130)	334 (168)	415 (273)	396 (255)	0.48 (0.50)
	Semantically related-Identical	26^+^	17	34*	41*	62**	0.05
	Unrelated-Semantically related	23*	33**	42***	47**	28*	−0.07*

*Note*. The Identical-Semantically related differences represent the cost to processing of having a semantically related preview relative to an identical preview. The Unrelated-Semantically related differences represent the semantic preview benefit associated with having a semantically related preview relative to an unrelated preview.

+ *p* < .10. *p < .05. **p < .01. ***p < .001.

All of the reading time measures were numerically shorter in the identical than the semantically related preview condition across grades, though these differences were not consistently statistically robust.

**Figure 4.**
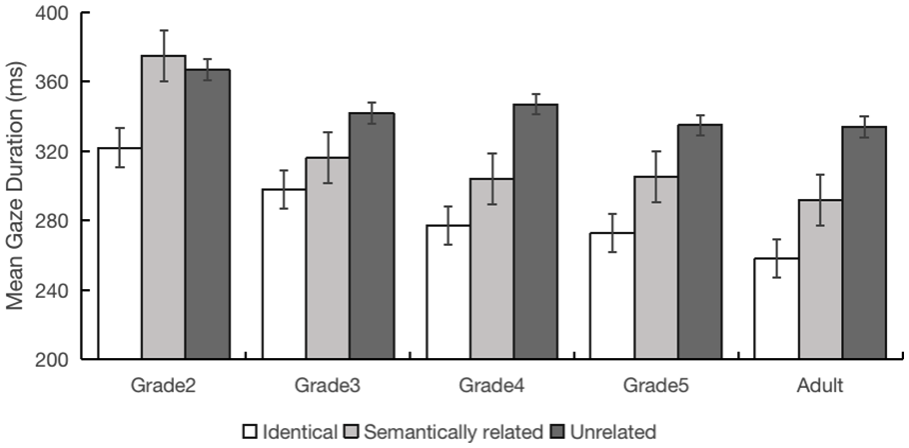
The semantic preview effects in gaze duration for the five participant groups (error bars represent standard errors of the mean).

In the measures of FFD and SFD, the semantic preview benefit effect was not significant for second, third and fourth grade students (*|t*s*|* < 1.46, *p*s > .05); however, this effect was robust in the fifth grade students and adults (*|t*s*|* > 2.52, *p*s < .05). For GD, second grade students showed no semantic preview benefit (*b* = −0.03, *SE* = 0.04, *t* = −0.62, *p* = .53), but third, fourth and fifth grade students as well as adults did show robust semantic preview benefit effects (*|t*s*|* > 2.23, *p*s < .05) (see Figure 4). There were no significant semantic preview benefit effects for second and third graders for go-past time (*|t*s*|* < 1.74, *p*s > .05), while this effect was significant across fourth and fifth graders, and adults (*|t*s*|* > 2.66, *p*s < .01). Only adult readers showed semantic preview benefit effects for total reading time (*b* = 0.09, *SE* = 0.04, *t* = 2.44, *p* = .01), whilst readers from the other groups did not show this effect (*|t*s*|* < 0.99, *p*s > .05). In skipping probability, there were no significant preview benefit effects for second, third and fourth graders (*|t*s*|* < 0.99, *p*s > .05), but a semantic preview benefit effect did occur in skipping for both fifth grade students and adults (*|t*s*|* > 2.37, *p*s < .05).

The results from Experiment 3 are less consistent across all measures than those from Experiment 2, in that the effects reflecting change in parafoveal preview benefit across grades derive from the gaze duration and go-past time measures. Nonetheless, based on the gaze duration measure, it appears that whilst participants from Grade 2 did not obtain a semantic preview benefit from the upcoming character, participants in the older age groups did. The gaze duration results (and less robustly, the go-past time results) suggest that effective semantic processing of an upcoming Chinese character develops and maintains from the third grade.^6^

### Discussion

Experiment 3 explored whether children from Grade 2 to Grade 5 (and adults) show semantic preview benefit for upcoming Chinese characters during natural Chinese reading. We undertook global analyses to measure developmental progression with age, and in our local analyses, we probed parafoveal processing through preview benefit effects for our target characters. Consistent with Experiments 1 and 2, our global analyses showed that readers’ total reading times, average fixation duration and fixation count reduced, while the rightward saccade extent did not show clear numerical trends with grade. These results are consistent with a substantial number of existing studies investigating Chinese and other language reading (e.g., Johnson et al., 2018; Milledge et al., 2021, 2022; Pagán et al., 2016; Tiffin-Richards & Schroeder, 2015; W. Zhou et al., 2018). Our local analyses showed that semantic preview benefit effects occurred from the third grade and maintained through to adults across our participant groups. However, these effects were only significant across these age groups for the gaze duration measure, with preview effects in first fixation and SFD occurring reliably only in the fifth grade and adult participants. Preview effects in the go-past reading time measure occurred at and beyond the fourth grade, with only adults showing robust preview effects in total reading time for the target character. Finally, target character skipping showed preview beneficial effects for fifth grade children and adults alone. Three points are noteworthy from the local analyses. First, semantic preview benefit effects appear to be developmentally delayed relative to orthographic preview benefit effects observed in Experiment 1. In relation to the phonological preview benefit effects observed in Experiment 2, the first signs of such effects were distributed across ages for different measures. These effects were observed in third-grade children in the gaze duration measure, but did not appear in the first fixation and single fixation measures until the fifth grade. To this extent, the onset of the effects was more distributed across the measures. It appears that there was little sensitivity to the semantic characteristics of the upcoming character during the first fixation on the target character. However, when refixations on that character were included (as captured in the gaze duration measure), there was a sensitivity to such information. Thus, it appears that a refixation on the target character was a pre-requisite for the semantic preview benefit effects to be observed (at least in relation to the first pass reading time measures and from the third grade and beyond). We attempted to quantify this difference by subtracting the first fixation measure from the gaze duration measure (thereby isolating the time spent refixating the target); however, because the target region was a single character, most of the resulting values were zero (90%), and we obtained no statistically reliable effects.

Given that semantic preview benefit effects have a contentious place in the eye movement literature, it is worth considering why the effects might have occurred so robustly in Experiment 3. First, the targets in the present experiment were single characters, meaning that they were small and pre-target fixations would be close to upcoming information. Second, written Chinese is unspaced, meaning that it is less horizontally spatially extended than word-spaced languages, thereby increasing the availability of parafoveal information. Third, written Chinese is a linguistically dense orthography with substantial information being conveyed by a character. Again, this density increases the possibility of obtaining semantic preview effects. Finally, it is also the case that Chinese is a logographic language, and the characters that comprise the orthography can be divided into two types: integrated characters and compound characters. Several studies have shown that in Chinese reading, adult readers can obtain semantic information from parafoveal regions regardless of whether the target character and preview character are integrated or compound characters (e.g., Yan et al., 2009, 2012). In order to ensure that the current experimental materials were broadly representative of the language, we ensured that the target characters in Experiment 3 were comprised of both integrated characters (12%) and compound characters (88%). And for the integrated characters, the mapping between orthography and meaning was close, thereby maximizing the chances of observing semantic preview effects (Yan et al., 2009). Furthermore, of the compound characters that formed the targets, over 72% were comprised of phonograms, the radicals of which can represent the meaning of the whole character. This is especially true of semantically transparent radicals, something that may also facilitate the acquisition of semantic information from a parafoveal character (Yan et al., 2012). Furthermore, it was also the case that approximately 28% of our target characters were ideographic, that is, formed from a combination of two or three radicals to show their meaning (e.g., the character “森” meaning *forest* is comprised of three radicals, each of the form “木”, itself a character that means *tree*. Thus, the meaning of the Chinese character “森” is directly derivable from its constituent radicals. Thus, the close connection between the orthographic and semantic characteristics of our target characters could have contributed to our observation semantic preview benefit effects.

The second noteworthy point from the local analyses concerns the effects of the later go past and total reading time measures. As with the earlier experiments, preview benefit effects in these measures were less stable and consistent across our participant groups, and we strongly suspect that this is due to these measures including fixations made both during first pass reading and re-reading of the sentence. The inclusion of second-pass fixations in these measures likely adds noise to the short-lived preview effects that occur when the target character is processed for the first time during sentence reading. Finally, we note that semantic preview effects in target character skipping appeared, developmentally, relatively late (fifth grade and adults), though we note also that these effects were coincident with the appearance of the effects in the first fixation and single fixation measures.

Overall, the results of Experiment 3 are, to some degree at least, similar to those of Experiment 2 in that the appearance of semantic preview benefit effects, like the appearance of phonological preview benefit effects, was delayed relative to the occurrence of orthographic preview benefit effects (Experiment 1).^7^ Assuming that orthographic preview effects are caused by the visual characteristics of parafoveal text, whereas phonological and semantic preview benefit effects are driven by the linguistic characteristics of text, together the results indicate that linguistic parafoveal processing has a delayed developmental trajectory relative to parafoveal processing that has as its basis visual processing. We will return to this issue in the General Discussion.

## General discussion

In three experiments, we investigated the development of orthographic, phonological and semantic parafoveal processing during Chinese natural reading. Children from second grade to fifth grade, as well as adults, read sentences with previews manipulated using the boundary paradigm. Our results showed a number of basic findings. First, we demonstrated that reading performance as indexed by a range of global eye movement measures improved with development. Basic developmental effects occurred in all three experiments (i.e., three replications of these developmental effects). Similar effects have been shown in previous studies (Blythe et al., 2011; Milledge et al., 2021; Pagán et al., 2016; Tiffin-Richards & Schroeder, 2015; Zang et al., 2013). As children become more proficient readers, they make fewer and shorter fixations and take less time generally to read sentences. The clarity and consistency of these results provide confidence that our experiment worked in the way that we anticipated and that our participant groups behaved as we expected (both in and of themselves, as well as in relation to each other).

Our second important finding was that children do engage in parafoveal processing of upcoming text during natural Chinese reading. We actively manipulated the nature of the information that was parafoveally available to readers in order to assess the influence of different previews. Based on the results of all three experiments, it is clear that children as young as second-grade readers demonstrate parafoveal preview benefit. The present study is the first to use the boundary paradigm to investigate preview benefit effects in children reading Chinese. To date, a small number of studies exist in the literature that have used the boundary paradigm to investigate preview effects in children reading alphabetic languages (Häikiö et al., 2010; Johnson et al., 2018; Marx et al., 2015, 2016; Milledge et al., 2021, 2022; Pagán et al., 2016; Tiffin-Richards & Schroeder, 2015). All of these studies showed that developing readers are able to process information from the parafovea during first-pass reading. Consistent with the present study, all these experiments also demonstrated that parafoveal processing is less effective in younger children, particularly relative to adult performance.

It is important to note that four of the alphabetic studies, Milledge et al. (2021, 2022), Pagán et al. (2016), and Tiffin-Richards and Schroeder (2015), compared a single group of similarly aged child readers with a group of adult readers. Consequently, these studies are not particularly informative with respect to children’s developmental change in reading across age. The only study to date that has assessed reading performance across a range of ages in children is that of Johnson et al. (2018), who used a single group of 48 children aged between 6.4 and 12.8 years. In their study, Johnson et al. manipulated the predictability and parafoveal availability of a word (previews were either visually similar or visually dissimilar to the target word) and carried out analyses that included age as a variable, as well as independent analyses that included reading fluency and decoding skill as variables. Thus, to date, Johnson et al.’s study provides the only experimental data that offer insight into the nature of children’s developmental change with age in parafoveal processing and eye movement behavior during reading. Very interestingly, Johnson et al. demonstrated that the preview benefit varied systematically with age and reading fluency. Older, more fluent readers showed greater preview benefit effects than younger, less fluent readers. This result led Johnson et al. to conclude that age and reading fluency were important determinants of the amount of (orthographic) information a child obtains from the parafovea during natural reading.

The present study extends these findings in a number of important ways. First, note that the present study investigated the development of parafoveal processing during reading in a large number of participants (366 child participants and 90 adults) across a wide age range (Grades 2–5, as well as a group of adults). Thus, the present study offers a quite comprehensive and detailed perspective on the development of parafoveal processing from an early stage of reading through to adult performance. Additionally, the relatively large number of participants in the present experiments ensures a good level of statistical power in relation to the effects that we probed at every age across the range that we sampled. Second, the present study investigated parafoveal processing in natural Chinese reading, that is, Chinese reading without any secondary task (c.f., W. Zhou et al., 2018). Thus, the present study investigated natural parafoveal processing during reading in a language that has not hitherto been examined. To this extent, the current findings are novel. Furthermore, similar to Johnson et al., in all three of the experiments reported here, we too found that preview benefit effects were greater in older compared with younger child readers. At a relatively gross level, it is clear that the present study offers an independent demonstration of the basic findings obtained by Johnson et al. in a language other than English, and our results add general support for the claim of increased availability of parafoveal information in older relative to younger child readers.

Next, let us consider the results from the present Experiment 1 in which we adopted an orthographic parafoveal preview manipulation across age. These results are most directly related to findings from Johnson et al.’s study, as they too adopted a manipulation of orthographic preview. An important aspect of the present results is that the orthographic preview effects obtained in Experiment 1 were relatively stable across all the participant groups in our study (e.g., in gaze duration, the preview effect was 24, 32, 23, 19, and 34 ms in Grade 2, 3, 4, 5, and adults, respectively). This stability contrasts with the effects reported by Johnson et al., who showed that orthographic preview benefit effects were smaller and less robust in younger relative to older readers. We suspect that the increased magnitude of effects in the younger readers and the relative stability of the effects across age in the present study relative to those of Johnson et al. likely arose because we adopted single-character parafoveal preview manipulations in the present experiments. Since Chinese is an orthographically dense, unspaced language (see Liversedge et al., 2016), single-character orthographic preview manipulations are relatively powerful, being close to the point of fixation and involving a significant proportion of visual change to a word (most words in Chinese are one or two characters in length). All these factors will have served to ensure substantial preview effects. Further, as noted earlier, we tested a relatively large number of participants in each age group (and we also used a relatively large number of stimuli in each experiment). These factors also, likely, contributed to the relatively large and stable orthographic parafoveal preview effects that we obtained across age in our study. With these suggestions in mind, it appears that, in fact, there is significant congruence between the present findings and those of Johnson et al.

Let us now turn to the results of Experiments 2 and 3, and in so doing turn our attention to a further line of theoretical investigation of the present study, that is, whether children showed a differential developmental time course in respect of the efficacy of parafoveal processing in relation to three qualitatively different forms of preview: orthographic, phonological and semantic previews. Recall, we predicted that phonological and semantic parafoveal processing might be dependent on effective orthographic processing, and therefore, we might expect that the development of effective orthographic parafoveal processing might occur at a younger age than effective phonological and semantic processing. Further, more tentatively, we suggested that effective phonological processing might develop prior to effective semantic processing, possibly due to the phonological form of a word being representationally less rich than its semantic form.

In fact, to quite a degree, our results patterned as we expected. In the early reading time measures of processing (first and SFD, and gaze duration) for which we might expect to see preview benefit effects, effects of phonological and semantic preview benefit were delayed in the younger children relative to the orthographic preview benefit effects. Recall that orthographic preview benefit effects occurred in even the youngest children. In contrast, phonological preview benefit effects were not apparent in these measures until the fourth grade; that is, phonological preview effects were developmentally delayed relative to orthographic preview benefit effects. Similarly, semantic preview benefit effects were delayed relative to orthographic preview benefit effects. Semantic preview benefit did not occur in the second-grade children, though these effects did appear solely in the gaze duration measure for the third- and fourth-grade children. More generally, by the fifth grade, orthographic, phonological and semantic preview benefit effects were observed across all the early reading time measures in a manner quite comparable to adult performance. Thus, in line with our experimental hypotheses, the developmental trajectory for phonological and semantic parafoveal processing was delayed relative to that for orthographic parafoveal processing. These results fit well with the suggestion that since the phonological and semantic characteristics of a written word are accessed via the word’s orthographic code, orthographic parafoveal processing might take developmental precedence over phonological and semantic parafoveal processing.

Before closing, it is important to note aspects of our results that were unexpected, to acknowledge one or two limitations of the present study and consider potential future directions for research that will develop understanding in this area. The only aspect of the developmental results that did not align with our a priori theorizing relates to our obtaining evidence that semantic and phonological parafoveal processing appear, developmentally, in children of approximately a similar age. Indeed, if anything, it might be argued that the evidence for semantic parafoveal processing appears in children who are slightly younger (Grade 3 and Grade 4 in the gaze duration measure) than for phonological processing (Grade 4 in first and SFD and gaze duration measures). Recall that we were tentative in our suggestion that the development of phonological parafoveal processing might precede the development of semantic parafoveal processing. It appears that this suggestion is incorrect.

In a cross-linguistic study, Feng et al. (2001) found that early phonological activation (e.g., FFD) existed in English reading, but not in Chinese reading. Feng et al. suggested that Chinese reading may differ from English reading in relation to the time course of phonological processing. This difference in processing might occur because phonological information may be activated through orthography directly, early, and automatically in English reading (Grainger & Holcomb, 2010, see also Tiffin-Richards, 2024 for early orthographic parafoveal processing in bilingual children’s and adults’ reading). This is not the case, however, in relation to semantic information where the linkage with orthography is much less direct. Relatedly, in Chinese reading, Yan et al. (2009) reported early semantic preview benefit (i.e., effects that occurred in FFD), but no corresponding early phonological preview benefit was obtained. Additionally, robust phonological preview benefit occurred in a slightly later measure (gaze duration). These results are consistent with the suggestion that in Chinese reading, semantic information may be extracted from the parafovea earlier than phonological information, at least with respect to adult readers processing relatively simple text (Yan et al.’s average target character frequency was over 1,000 per million, quite high, and the average number of strokes per character was 5, quite low). These findings support the suggestion that readers gain direct access to semantics from orthography (as per Dual-Route theories of Chinese character recognition, e.g., X. Zhou & Marslen-Wilson, 1996; X. Zhou et al., 1999). In the present study, we purposefully developed our experimental materials to ensure that they were appropriate for Grade 2 students. This meant that our stimuli were relatively easy to read and for such stimuli, semantic information may be particularly readily accessible via orthography. This may be considered a limitation of the current work, and quite whether similar effects might occur for linguistic stimuli that are more demanding is an empirical question for future research.

It is also important to reiterate that in all three of the experiments that we report here, we manipulated preview effects in relation to a single parafoveal character. We did this in order to maximize the possibility of obtaining preview benefit effects, particularly in younger children. Indeed, our manipulations were successful in inducing the anticipated effects, even in relation to semantic parafoveal preview manipulations. We are convinced that our use of single-character preview manipulations contributed significantly to our obtaining such strong effects in the experiments. We note that most words in Chinese are one or two characters long; however, there are quite a number of words (or multi-constituent units, MCUs; Zang, 2019) that are three or four characters long. To some, the fact that we manipulated only the first parafoveal character in our study may also be considered a limitation, as it remains an empirical question as to whether the effects we obtained here generalize to characters beyond one parafoveal character. However, we consider that this will very likely be the case, particularly in light of recent research demonstrating that longer words and phrases (3–4 characters in length) and MCUs (e.g., idioms and other multi-word expressions) are processed parafoveally and foveally as single elements (see Zang et al., 2021; Zang, Fu, et al., 2024; Zang, Wang, et al., 2024; see also Zang, 2019). Thus, we strongly suspect that the effects we report here will generalize beyond words of a single character, and we consider future studies to investigate such a possibility to be a priority. What is evident from the current results, however, is that for preview manipulations of the upcoming character, it is clearly possible to demonstrate semantic parafoveal preview benefit effects, and further, that these effects are apparent in relatively young children.

A final point worth consideration concerns the generality of developmental effects across eye movement measures. In our discussion thus far, we have focused primarily on the earliest reading time measures of processing associated with the identification of a word, or character, that is, first and SFD and gaze duration. We have done this because these measures are known to reflect influences from parafoveal manipulations. However, we also computed other measures from the eye movement record, namely, skipping rates alongside go past and total reading times. The very striking consideration in relation to these measures is that the patterns of effects across the age groups are quite unstable. Character skipping effects appear intermittently and inconsistently across the experiments. Similarly, effects in total reading time and go past reading times, whilst showing a general trend of preview benefit being more apparent in the older age groups relative to the younger age groups, are, nonetheless, inconsistent. We can probably conclude two things on the basis of this aspect of the results. First, in relation to go past and total reading times, since these measures include fixations that are made after the word is initially encountered, then it is likely that these later fixations reflect processing other than that associated with parafoveal preview manipulations. Thus, these fixations will likely dilute any preview benefit effects that were apparent in the earlier measures. It is, therefore, unsurprising that effects in these later reading time measures are somewhat inconsistent relative to the effects obtained in the earlier measures. Second, it is very clear that there is far less consistency both across the range of ages, and across the three experiments, in the skipping data relative to the reading time data (though note that the most robust skipping effects occurred for the oldest children and the adults). This aspect of the results strongly suggests that parafoveal linguistic processing exerts an earlier and stronger influence over oculomotor decisions of when to move the eyes relative to decisions regarding where to move the eyes. Dissociations between “when” and “where” aspects of oculomotor control are well established (Radach & Heller, 2000; Rayner, 2009; Rayner & McConkie, 1976; Rayner & Pollatsek, 1981; Reichle et al., 2011), and it is, therefore, not so surprising that there might also exist differences in their developmental ontogenesis. Again, it is our view that future studies investigating the development of parafoveal processing during reading might report later reading time measures as well as more “standard,” earlier, measures in order that we develop our understanding of the time course of parafoveal processing effects and how such effects are modulated by later aspects of processing in reading.

In summary, the present study provides the first data to reveal the developmental time course of parafoveal processing in Chinese children’s reading. The three experiments that we report here replicate basic findings associated with developmental change in eye movement control during reading. Finally, we showed that orthographic parafoveal processing develops earlier than phonological and semantic parafoveal processing during Chinese reading. Together, the results provide a quite comprehensive picture of how a process that is fundamental to reading in Chinese and other languages develops with age.

## Supplemental Material

sj-docx-1-qjp-10.1177_17470218251372482 – Supplemental material for Development of orthographic, phonological and semantic parafoveal processing in Chinese reading Supplemental material, sj-docx-1-qjp-10.1177_17470218251372482 for Development of orthographic, phonological and semantic parafoveal processing in Chinese reading by Min Liu, Sainan Li, Zhu Meng, Yongsheng Wang, Chuanli Zang, Guoli Yan and Simon P Liversedge in Quarterly Journal of Experimental Psychology

## Notes

1. Although the experiments discussed earlier involved manipulations of orthography (e.g., letter transpositions), such manipulations also often produced changes in phonological characteristics of parafoveal stimuli. Nonetheless, primary in those studies was the nature of parafoveal orthographic processing.

2. All the target words in Experiments 1 to 3 were two characters long apart from three stimuli in Experiment 2. Two of these stimuli were single-character words and the other was the second character of a two-character word. We conducted all analyses below with and without these stimuli and the pattern of results was identical. Analyses below include all stimuli.

3. In Experiment 1, we also analyzed a more complex model including random slopes. The results were the same as those with the simplest model. In Experiment 3, reported subsequently, only the simplest model converged for all the measures, therefore, for consistency, we report the results from the simplest model in all three experiments.

4. We computed how often the pre-target word was a single character word in each of the experiments. It was the case that the proportion was roughly equivalent in all three experiments; 67% of the pre-target words were one-character words in Experiment 1, 55% of the pre-target words are one-character words in Experiment 2, and 62% of the pre-target words are one-character words in Experiments 3. Preview effects can often be a function of the previous fixation location. To investigate this possibility, we analyzed launch site as a continuous independent variable in Experiments 1, 2, and 3. The results showed that there was very little modulatory effect of this variable in the analyses of preview for each experiment. For Experiment 1, there was one two-way interaction and one three-way interaction out of 18 analyses. Neither effect was large, nor systematic. For Experiment 2 there was a significant interaction between phonological preview benefit and launch site for Grades 2 and 3, however, these effects are clearly meaningless because the second and third graders did not show phonological preview effects. The interaction between phonological preview benefit and the launch site for Grade 4 in SFD (but no other measures) was marginal (*b* = 0.04, *SE* = 0.02, *t* = 1.92, *p* = .05). No other effects were significant for Experiment 2. Finally, for Experiment 3 there were no significant effects (see Supplemental Material for analyses in full). We interpret the full set of statistical tests as demonstrating that there were no meaningful effects of launch site on the preview benefit effects we observed across all three experiments.

5. In order to assess whether there were differential frequency differences between the related previews, unrelated previews and target words (within items) in Experiments 1, 2, and 3 (between items), we conducted a 3 × 3 mixed ANOVA. None of the effects were reliable (all *F* < 2.25, *p* > .10), indicating that there were no differential effects of frequency in our stimuli across conditions or experiment. Furthermore, in order to be confident that frequency did not contribute to our effects, we undertook a new set of LMM analyses in which we included frequency as a covariate. These analyses provided results that were entirely consistent with our original results, further suggesting that we were correct to attribute our effects to differences in processing of orthographic, phonological and semantic previews at different ages (rather than the effects arising due to frequency differences). These analyses are reported in full on the OSF website.

6. A type II Wald χ^2^ test was conducted using the Anova function of the car package (Version 3.1-2) in R (Fox et al., 2013) to examine the overall effects of preview, grade and their interactions. These results were entirely in accord with the results for our LMMs across three experiments. Furthermore, we undertook analyses in which we used the glht function of package multcomp in R to control for the multiple comparisons (Hothorn et al., 2008). The patterns of results were the same for Experiments 1 and 2, and whilst there were a small number of minor differences in the significance of effects for Experiment 3, none of these undermined the theoretical conclusions offer here. For the interested reader, the analyses corrected for multiple comparisons are reported in full on the OSF website.

7. We did not test the children to assess all three types of parafoveal processing in a single experiment because a single experiment of this form would have been too long and would have represented too much of a challenge for them. An experiment of a long duration (e.g., 1.5 hr testing) would have caused the children to experience discomfort which would be unacceptable. Furthermore, it would have resulted in poor quality eye movement data. Our approach in the present experiment accords well with that adopted in the vast majority of other eye tracking experiments investigating children’s reading (e.g., Tiffin-Richards & Schroeder, 2015). Furthermore, we undertook a single formal statistical analysis in which we evaluated the preview benefit for each of the three experiments across the grades (and the adults) that we tested. These results were consistent with our claims. The full analysis and graphical representations of the effects are available at https://osf.io/zj3un/?view_only=38a9db3f32534487b91c3aef2bb02045.
