# Supplementary material for: Development of orthographic, phonological and semantic parafoveal processing in Chinese reading
Source: Q J Exp Psychol (Hove). 2025 Aug 22;79(4):905–27. doi: 10.1177/17470218251372482 (PMC12982572; doi:10.1177/17470218251372482)
Supplement: sj-docx-1-qjp-10.1177_17470218251372482 – Supplemental material for Development of orthographic, phonological and semantic parafoveal processing in Chinese reading [file sj-docx-1-qjp-10.1177_17470218251372482.docx]

Supplementary Material for:

**Development of Orthographic, Phonological and Semantic Parafoveal Processing in Chinese Reading**

Min Liu^1,2^, Sainan Li^3^, Zhu Meng^4^, Yongsheng Wang^1^, Chuanli Zang^5^,

Guoli Yan^1^ & Simon P. Liversedge^6,7^

1 Key Research Base of Humanities and Social Sciences of the Ministry of Education, Tianjin Social Science Laboratory of Students' Mental Development and Learning, Faculty of Psychology, Tianjin Normal University

2 School of Educational Sciences, Liaocheng University

3 Institute of Moral Education and Educational Psychology, Tianjin Academy of Educational Sciences

4 School of Educational Sciences, Jiangsu Normal University

5 School of Psychology, Liverpool Hope University

6 School of Psychology and Humanities, University of Lancashire

7 Northumbria University

Corresponding Author:

Zang, Chuanli

Liverpool Hope University

Liverpool L16 9JD

UK

<https://orcid.org/0000-0002-9573-4968>

All the data sets and R analysis code of the current study are available at <https://osf.io/zj3un/?view_only=38a9db3f32534487b91c3aef2bb02045>

Funding source: We acknowledge support from ESRC Grant (ES/R003386/1), Major Project of the Key Research Base of Humanities and Social Sciences of the Ministry of Education (22JJD190012), a scholarship from the China Scholarship Council, and Doctoral Scientific Research Foundation of Liaocheng University (321052006).

**Development of Orthographic, Phonological and Semantic Parafoveal Processing in Chinese Reading**

**Additional Analyses**

**Contents**

1. The orthographic preview benefit and preview cost for Experiment 1.
2. The phonological preview benefit and preview cost for Experiment 2.
3. The semantic preview benefit and preview cost for Experiment 3.
4. LMM analyses on the target when launch site was included as a continuous variable in Experiments 1-3.
5. **The orthographic preview benefit and preview cost for Experiment 1**

On the target character of Experiment 1, we also analyzed the main effects of grade and preview, and the interactions between orthographic preview benefit and grade. The summary statistics for all the measures are provided in Table S1.

There was a numerical tendency toward reduced reading times and an increased skipping rate with grade, however, the main effect of grade was not significant across FFD, SFD, GD, go-past time and skipping probability measures (|*ts*| < 1.36, *ps* > 0.05). Target character total reading times for second graders were significantly longer than for third graders (*b* = -0.12, *SE* = 0.05, *t* = -2.28, *p* = 0.02), and in turn were significantly longer for third than fourth graders (*b* = -0.16, *SE* = 0.05, *t* = -3.09, *p* < 0.01). This difference was not statistically robust for fourth compared with fifth graders (*b* = -0.03, *SE* = 0.05, *t* = -0.54, *p* = 0.59), but it was significant for fifth graders compared with adults (*b* = -0.13, *SE* = 0.05, *t* = -2.51, *p* = 0.01). Across all the reading time measures, there was a main effect of preview (|*ts*| > 2.67, *ps* < 0.05), with the shortest times under the identical preview condition and the longest times under the unrelated preview condition.

The interactions associated with the orthographic preview benefit (related – unrelated preview) across grades were not significant across all the reading time measures (|*ts*| < 1.46, *ps* > 0.05), indicating that orthographic preview benefit effects for the target character were comparable across all the grades.

To quantify the evidence for the null interaction, we used the Bayes Factor package (version 0.9.12 - 4.2; Morey et al., 2015, available in R) to compute Bayes Factors comparing the model without an interaction against the model that included an interaction. We found that the null hypotheses, that is, the likelihood that the models without an interaction were at least 24, 20, 30, 12 and 39 times more likely to be true than the alternative models including the interaction for FFD, SFD, GD, go-past time and TD, respectively. Therefore, the models without the interaction terms were favored and our findings provide evidence in favor of the null. The null interaction between the orthographic preview benefit effect and grade across all the reading time measures suggests that the orthographic preview benefit was comparable across grades. The results show that the ability to pre-process orthographic information from upcoming characters is in place by the second grade and remains steady until adulthood.

Table S1. Results of Linear Mixed Effects on the Target Across Conditions

|  | *β* | *SE* | \|*t*\| or \|*z*\| | *p* |
| --- | --- | --- | --- | --- |
| **First fixation** |  |  |  |  |
| Intercept | 5.47 | 0.01 | 372.67 | < 0.001 |
| Grade 2 vs. Grade 3 | -0.02 | 0.04 | 0.58 | 0.56 |
| Grade 3 vs. Grade 4 | -0.02 | 0.04 | 0.37 | 0.71 |
| Grade 4 vs. Grade 5 | 0.01 | 0.04 | 0.13 | 0.90 |
| Grade 5 vs. Adult | -0.03 | 0.04 | 0.58 | 0.56 |
| Preview cost | 0.06 | 0.02 | 3.47 | 0.001 |
| Orthographic preview benefit | 0.08 | 0.02 | 5.14 | < 0.001 |
| Preview cost * Grade (G2-G3) | 0.06 | 0.05 | 1.25 | 0.21 |
| Preview cost * Grade (G3-G4) | -0.01 | 0.05 | 0.13 | 0.90 |
| Preview cost * Grade (G4-G5) | 0.02 | 0.05 | 0.29 | 0.77 |
| Preview cost * Grade (G5-Adult) | -0.01 | 0.05 | 0.26 | 0.79 |
| Orthographic preview benefit * Grade (G2-G3) | -0.03 | 0.05 | 0.51 | 0.61 |
| Orthographic preview benefit * Grade (G3-G4) | 0.01 | 0.05 | 0.16 | 0.87 |
| Orthographic preview benefit * Grade (G4-G5) | -0.02 | 0.05 | 0.34 | 0.73 |
| Orthographic preview benefit * Grade (G5-Adult) | 0.03 | 0.05 | 0.51 | 0.61 |
| **Single fixation** |  |  |  |  |
| Intercept | 5.47 | 0.02 | 358.45 | < 0.001 |
| Grade 2 vs. Grade 3 | -0.02 | 0.04 | 0.43 | 0.67 |
| Grade 3 vs. Grade 4 | -0.00 | 0.04 | 0.11 | 0.91 |
| Grade 4 vs. Grade 5 | -0.01 | 0.04 | 0.14 | 0.89 |
| Grade 5 vs. Adult | -0.02 | 0.04 | 0.46 | 0.64 |
| Preview cost | 0.06 | 0.02 | 3.35 | < 0.001 |
| Orthographic preview benefit | 0.09 | 0.02 | 5.14 | < 0.001 |
| Preview cost * Grade (G2-G3) | 0.05 | 0.05 | 0.89 | 0.37 |
| Preview cost * Grade (G3-G4) | 0.00 | 0.05 | 0.08 | 0.93 |
| Preview cost * Grade (G4-G5) | 0.01 | 0.06 | 0.23 | 0.82 |
| Preview cost * Grade (G5-Adult) | -0.02 | 0.06 | 0.29 | 0.77 |
| Orthographic preview benefit * Grade (G2-G3) | -0.00 | 0.05 | 0.08 | 0.94 |
| Orthographic preview benefit * Grade (G3-G4) | -0.00 | 0.05 | 0.05 | 0.96 |
| Orthographic preview benefit * Grade (G4-G5) | -0.01 | 0.05 | 0.28 | 0.78 |
| Orthographic preview benefit * Grade (G5-Adult) | 0.02 | 0.05 | 0.43 | 0.67 |
| **Gaze duration** |  |  |  |  |
| Intercept | 5.51 | 0.02 | 324.17 | < 0.001 |
| Grade 2 vs. Grade 3 | -0.05 | 0.05 | 1.01 | 0.31 |
| Grade 3 vs. Grade 4 | -0.03 | 0.05 | 0.52 | 0.61 |
| Grade 4 vs. Grade 5 | -0.01 | 0.05 | 0.14 | 0.89 |
| Grade 5 vs. Adult | -0.03 | 0.05 | 0.66 | 0.51 |
| Preview cost | 0.08 | 0.02 | 4.57 | < 0.001 |
| Orthographic preview benefit | 0.09 | 0.02 | 5.11 | < 0.001 |
| Preview cost * Grade (G2-G3) | 0.03 | 0.05 | 0.51 | 0.61 |
| Preview cost * Grade (G3-G4) | -0.02 | 0.06 | 0.27 | 0.79 |
| Preview cost * Grade (G4-G5) | 0.05 | 0.06 | 0.81 | 0.42 |
| Preview cost * Grade (G5-Adult) | -0.04 | 0.06 | 0.70 | 0.49 |
| Orthographic preview benefit * Grade (G2-G3) | 0.01 | 0.05 | 0.19 | 0.85 |
| Orthographic preview benefit * Grade (G3-G4) | -0.02 | 0.06 | 0.33 | 0.74 |
| Orthographic preview benefit * Grade (G4-G5) | -0.02 | 0.06 | 0.31 | 0.76 |
| Orthographic preview benefit * Grade (G5-Adult) | 0.03 | 0.06 | 0.59 | 0.55 |
| **Go-past duration** |  |  |  |  |
| Intercept | 5.79 | 0.02 | 246.76 | < 0.001 |
| Grade 2 vs. Grade 3 | -0.04 | 0.07 | 0.65 | 0.51 |
| Grade 3 vs. Grade 4 | -0.09 | 0.07 | 1.35 | 0.18 |
| Grade 4 vs. Grade 5 | -0.08 | 0.07 | 1.19 | 0.24 |
| Grade 5 vs. Adult | -0.02 | 0.07 | 0.28 | 0.78 |
| Preview cost | 0.07 | 0.03 | 2.68 | 0.01 |
| Orthographic preview benefit | 0.16 | 0.03 | 5.90 | < 0.001 |
| Preview cost * Grade (G2-G3) | -0.00 | 0.08 | 0.01 | 0.99 |
| Preview cost * Grade (G3-G4) | 0.00 | 0.08 | 0.05 | 0.96 |
| Preview cost * Grade (G4-G5) | -0.04 | 0.09 | 0.43 | 0.67 |
| Preview cost * Grade (G5-Adult) | -0.03 | 0.09 | 0.30 | 0.77 |
| Orthographic preview benefit * Grade (G2-G3) | -0.01 | 0.08 | 0.09 | 0.93 |
| Orthographic preview benefit * Grade (G3-G4) | -0.04 | 0.08 | 0.47 | 0.64 |
| Orthographic preview benefit * Grade (G4-G5) | -0.03 | 0.08 | 0.33 | 0.74 |
| Orthographic preview benefit * Grade (G5-Adult) | 0.09 | 0.09 | 1.10 | 0.27 |
| **Total duration** |  |  |  |  |
| Intercept | 5.77 | 0.02 | 299.76 | < 0.001 |
| Grade 2 vs. Grade 3 | -0.12 | 0.05 | 2.28 | 0.02 |
| Grade 3 vs. Grade 4 | -0.16 | 0.05 | 3.09 | < 0.01 |
| Grade 4 vs. Grade 5 | -0.03 | 0.05 | 0.54 | 0.59 |
| Grade 5 vs. Adult | -0.13 | 0.05 | 2.51 | 0.01 |
| Preview cost | 0.06 | 0.02 | 3.49 | < 0.001 |
| Orthographic preview benefit | 0.06 | 0.02 | 3.44 | < 0.001 |
| Preview cost * Grade (G2-G3) | 0.01 | 0.05 | 0.17 | 0.87 |
| Preview cost * Grade (G3-G4) | -0.01 | 0.06 | 0.13 | 0.90 |
| Preview cost * Grade (G4-G5) | -0.03 | 0.06 | 0.51 | 0.61 |
| Preview cost * Grade (G5-Adult) | 0.02 | 0.06 | 0.38 | 0.70 |
| Orthographic preview benefit * Grade (G2-G3) | 0.08 | 0.05 | 1.45 | 0.15 |
| Orthographic preview benefit * Grade (G3-G4) | -0.04 | 0.05 | 0.70 | 0.48 |
| Orthographic preview benefit * Grade (G4-G5) | 0.06 | 0.06 | 1.03 | 0.30 |
| Orthographic preview benefit * Grade (G5-Adult) | -0.05 | 0.06 | 0.87 | 0.39 |
| **Skipping probability** |  |  |  |  |
| Intercept | 0.39 | 0.07 | 5.48 | < 0.001 |
| Grade 2 vs. Grade 3 | 0.02 | 0.20 | 0.12 | 0.90 |
| Grade 3 vs. Grade 4 | 0.18 | 0.20 | 0.90 | 0.37 |
| Grade 4 vs. Grade 5 | -0.02 | 0.20 | 0.11 | 0.91 |
| Grade 5 vs. Adult | 0.11 | 0.20 | 0.58 | 0.57 |
| Preview cost | -0.15 | 0.06 | 2.52 | 0.01 |
| Orthographic preview benefit | -0.06 | 0.06 | 1.00 | 0.32 |
| Preview cost * Grade (G2-G3) | 0.01 | 0.19 | 0.08 | 0.94 |
| Preview cost * Grade (G3-G4) | 0.11 | 0.19 | 0.59 | 0.55 |
| Preview cost * Grade (G4-G5) | -0.34 | 0.19 | 1.79 | 0.07 |
| Preview cost * Grade (G5-Adult) | 0.10 | 0.19 | 0.54 | 0.59 |
| Orthographic preview benefit * Grade (G2-G3) | -0.02 | 0.19 | 0.10 | 0.92 |
| Orthographic preview benefit * Grade (G3-G4) | -0.28 | 0.19 | 1.49 | 0.14 |
| Orthographic preview benefit * Grade (G4-G5) | -0.16 | 0.19 | 0.85 | 0.39 |
| Orthographic preview benefit * Grade (G5-Adult) | 0.57 | 0.19 | 3.06 | < 0.01 |

Note: +*p* < 0.10, **p* < 0.05, ***p* < 0.01, ****p* < 0.001.

**Reference**

1. Morey, R. D., Rouder, J. N., & Jamil, T. (2015). Package “BayesFactor”. R package 0.9.12 - 4.2. https://cran.rproject.org/web/packages/BayesFactor/index.html

1. **The phonological preview benefit and preview cost for Experiment 2**

As shown in Table S2, on the target character of Experiment 2, there was a numerical tendency toward shorter reading times and an increased skipping probability with grade, however, the main effect of grade was not significant across FFD, SFD, GD, go-past time and skipping probability measures (|*ts*| < 1.64, *ps* > 0.05). The total reading times for second graders were numerically longer than for third graders (*b* = -0.09, *SE* = 0.05, *t* = -1.80, *p* = 0.07). The difference between third and fourth graders was not significant (*b* = -0.04, *SE* = 0.05, *t* = -0.84, *p* = 0.40), and in turn numerically longer for fourth than fifth graders (*b* = -0.09, *SE* = 0.05, *t* = -1.80, *p* = 0.07). This difference was statistically robust for fifth graders compared with adults (*b* = -0.11, *SE* = 0.05, *t* = -2.06, *p* = 0.04). Across all the reading time measures, there were main effects of preview (|*ts*| > 1.98, *ps* < 0.05), with the shortest times in the identical condition and the longest times in the unrelated preview conditions.

In addition, significant interactive effects for FFD, SFD and GD occurred in relation to the phonological preview benefit (related – unrelated preview) across grades (|*ts*| > 2.00, *ps* < 0.05). The pattern of results was such that robust preview benefit effects occurred for fourth and fifth graders as well as adults, but these effects were not robust for second and third grade participants. There were no significant interactive effects associated with go-past reading time (|*ts*| < 1.5, *ps* > 0.10). The interaction for total reading time approached significance consistent with a step change in preview benefit effects for fourth graders. Preview benefit effects were reduced for second and third grade children and these effects did not differ between these age groups (*b* = 0.01, *SE* = 0.05, *t* = 0.28, *p* = 0.78). Preview benefit increased significantly between the third and fourth grade children (*b* = 0.10, *SE* = 0.05, *t* = 1.93, *p* = 0.05), and maintained to the fifth grade children. There was no significant difference between fifth grade children and adults (*b* = -0.06, *SE* = 0.05, *t* = -1.21, *p* = 0.26). For skipping probability, the interaction did not achieve significance (*b* = -0.32, *SE* = 0.18, *z* = -1.81, *p* =0.07). For this measure, preview benefit effects were significant for second grade and adult readers, but not for the other grades (no significant difference between second, third, fourth and fifth graders, |*ts*| < 1.50, *ps* > 0.30).

Table S2. Results of Linear Mixed Effects on the Target Across Conditions

|  | *β* | *SE* | \|*t*\| or \|*z*\| | *p* |
| --- | --- | --- | --- | --- |
| **First fixation** |  |  |  |  |
| Intercept | 5.53 | 0.01 | 371.81 | < 0.001 |
| Grade 2 vs. Grade 3 | -0.01 | 0.04 | 0.17 | 0.87 |
| Grade 3 vs. Grade 4 | -0.03 | 0.04 | 0.80 | 0.43 |
| Grade 4 vs. Grade 5 | -0.04 | 0.04 | 0.94 | 0.35 |
| Grade 5 vs. Adult | 0.00 | 0.04 | 0.07 | 0.94 |
| Preview cost | 0.12 | 0.02 | 8.18 | < 0.001 |
| Phonological preview benefit | 0.03 | 0.01 | 2.35 | 0.02 |
| Preview cost * Grade (G2-G3) | 0.01 | 0.04 | 0.25 | 0.80 |
| Preview cost * Grade (G3-G4) | -0.07 | 0.05 | 1.37 | 0.17 |
| Preview cost * Grade (G4-G5) | 0.01 | 0.05 | 0.11 | 0.92 |
| Preview cost * Grade (G5-Adult) | 0.03 | 0.05 | 0.66 | 0.51 |
| Phonological preview benefit * Grade (G2-G3) | 0.00 | 0.04 | 0.01 | 0.99 |
| Phonological preview benefit * Grade (G3-G4) | 0.12 | 0.05 | 2.50 | 0.01 |
| Phonological preview benefit * Grade (G4-G5) | 0.01 | 0.05 | 0.30 | 0.76 |
| Phonological preview benefit * Grade (G5-Adult) | -0.03 | 0.05 | 0.68 | 0.50 |
| **Single fixation** |  |  |  |  |
| Intercept | 5.53 | 0.02 | 359.11 | < 0.001 |
| Grade 2 vs. Grade 3 | -0.01 | 0.05 | 0.18 | 0.86 |
| Grade 3 vs. Grade 4 | -0.03 | 0.05 | 0.59 | 0.56 |
| Grade 4 vs. Grade 5 | -0.05 | 0.05 | 1.18 | 0.24 |
| Grade 5 vs. Adult | 0.01 | 0.05 | 0.28 | 0.78 |
| Preview cost | 0.12 | 0.02 | 7.72 | < 0.001 |
| Phonological preview benefit | 0.03 | 0.02 | 1.99 | 0.05 |
| Preview cost * Grade (G2-G3) | 0.00 | 0.05 | 0.04 | 0.97 |
| Preview cost * Grade (G3-G4) | -0.05 | 0.05 | 1.04 | 0.30 |
| Preview cost * Grade (G4-G5) | -0.02 | 0.05 | 0.33 | 0.74 |
| Preview cost * Grade (G5-Adult) | 0.01 | 0.05 | 0.29 | 0.77 |
| Phonological preview benefit * Grade (G2-G3) | -0.02 | 0.05 | 0.35 | 0.73 |
| Phonological preview benefit * Grade (G3-G4) | 0.15 | 0.05 | 2.97 | < 0.01 |
| Phonological preview benefit * Grade (G4-G5) | -0.00 | 0.05 | 0.05 | 0.96 |
| Phonological preview benefit * Grade (G5-Adult) | -0.01 | 0.05 | 0.25 | 0.80 |
| **Gaze duration** |  |  |  |  |
| Intercept | 5.58 | 0.02 | 328.78 | < 0.001 |
| Grade 2 vs. Grade 3 | -0.01 | 0.05 | 0.27 | 0.79 |
| Grade 3 vs. Grade 4 | -0.03 | 0.05 | 0.70 | 0.48 |
| Grade 4 vs. Grade 5 | -0.05 | 0.05 | 1.06 | 0.29 |
| Grade 5 vs. Adult | -0.02 | 0.05 | 0.42 | 0.68 |
| Preview cost | 0.14 | 0.02 | 8.55 | < 0.001 |
| Phonological preview benefit | 0.04 | 0.02 | 2.61 | 0.01 |
| Preview cost * Grade (G2-G3) | 0.02 | 0.05 | 0.45 | 0.65 |
| Preview cost * Grade (G3-G4) | -0.07 | 0.05 | 1.41 | 0.16 |
| Preview cost * Grade (G4-G5) | -0.01 | 0.05 | 0.27 | 0.79 |
| Preview cost * Grade (G5-Adult) | 0.01 | 0.05 | 0.20 | 0.84 |
| Phonological preview benefit * Grade (G2-G3) | -0.00 | 0.05 | 0.06 | 0.96 |
| Phonological preview benefit * Grade (G3-G4) | 0.14 | 0.05 | 2.79 | 0.01 |
| Phonological preview benefit * Grade (G4-G5) | 0.04 | 0.05 | 0.71 | 0.48 |
| Phonological preview benefit * Grade (G5-Adult) | -0.04 | 0.05 | 0.75 | 0.45 |
| **Go-past duration** |  |  |  |  |
| Intercept | 5.86 | 0.02 | 255.95 | < 0.001 |
| Grade 2 vs. Grade 3 | -0.06 | 0.06 | 0.92 | 0.36 |
| Grade 3 vs. Grade 4 | -0.08 | 0.06 | 1.27 | 0.21 |
| Grade 4 vs. Grade 5 | -0.07 | 0.06 | 1.05 | 0.29 |
| Grade 5 vs. Adult | -0.10 | 0.06 | 1.54 | 0.13 |
| Preview cost | 0.19 | 0.02 | 7.90 | < 0.001 |
| Phonological preview benefit | 0.05 | 0.02 | 2.11 | 0.03 |
| Preview cost * Grade (G2-G3) | 0.01 | 0.07 | 0.20 | 0.84 |
| Preview cost * Grade (G3-G4) | -0.09 | 0.08 | 1.16 | 0.25 |
| Preview cost * Grade (G4-G5) | -0.05 | 0.08 | 0.69 | 0.49 |
| Preview cost * Grade (G5-Adult) | 0.05 | 0.08 | 0.62 | 0.54 |
| Phonological preview benefit * Grade (G2-G3) | 0.03 | 0.07 | 0.41 | 0.68 |
| Phonological preview benefit * Grade (G3-G4) | 0.10 | 0.07 | 1.37 | 0.17 |
| Phonological preview benefit * Grade (G4-G5) | 0.02 | 0.08 | 0.26 | 0.79 |
| Phonological preview benefit * Grade (G5-Adult) | -0.08 | 0.07 | 1.13 | 0.26 |
| **Total duration** |  |  |  |  |
| Intercept | 5.79 | 0.02 | 290.45 | < 0.001 |
| Grade 2 vs. Grade 3 | -0.09 | 0.05 | 1.80 | 0.07 |
| Grade 3 vs. Grade 4 | -0.04 | 0.05 | 0.84 | 0.40 |
| Grade 4 vs. Grade 5 | -0.09 | 0.05 | 1.80 | 0.07 |
| Grade 5 vs. Adult | -0.11 | 0.05 | 2.06 | 0.04 |
| Preview cost | 0.12 | 0.02 | 6.77 | < 0.001 |
| Phonological preview benefit | 0.05 | 0.02 | 2.97 | < 0.01 |
| Preview cost * Grade (G2-G3) | -0.05 | 0.05 | 0.93 | 0.35 |
| Preview cost * Grade (G3-G4) | -0.10 | 0.05 | 1.95 | 0.05 |
| Preview cost * Grade (G4-G5) | 0.04 | 0.05 | 0.68 | 0.50 |
| Preview cost * Grade (G5-Adult) | 0.06 | 0.06 | 1.07 | 0.29 |
| Phonological preview benefit * Grade (G2-G3) | 0.01 | 0.05 | 0.28 | 0.78 |
| Phonological preview benefit * Grade (G3-G4) | 0.10 | 0.05 | 1.93 | 0.05 |
| Phonological preview benefit * Grade (G4-G5) | 0.02 | 0.05 | 0.38 | 0.70 |
| Phonological preview benefit * Grade (G5-Adult) | -0.06 | 0.05 | 1.12 | 0.26 |
| **Skipping probability** |  |  |  |  |
| Intercept | 0.13 | 0.07 | 1.86 | 0.06 |
| Grade 2 vs. Grade 3 | 0.05 | 0.21 | 0.26 | 0.79 |
| Grade 3 vs. Grade 4 | 0.34 | 0.21 | 1.63 | 0.10 |
| Grade 4 vs. Grade 5 | -0.13 | 0.21 | 0.60 | 0.55 |
| Grade 5 vs. Adult | -0.00 | 0.21 | 0.01 | 0.99 |
| Preview cost | -0.11 | 0.06 | 1.94 | 0.05 |
| Phonological preview benefit | -0.21 | 0.06 | 3.67 | < 0.001 |
| Preview cost * Grade (G2-G3) | -0.21 | 0.18 | 1.16 | 0.25 |
| Preview cost * Grade (G3-G4) | 0.26 | 0.18 | 1.40 | 0.16 |
| Preview cost * Grade (G4-G5) | -0.25 | 0.18 | 1.37 | 0.17 |
| Preview cost * Grade (G5-Adult) | -0.02 | 0.18 | 0.12 | 0.90 |
| Phonological preview benefit * Grade (G2-G3) | 0.18 | 0.18 | 1.00 | 0.32 |
| Phonological preview benefit * Grade (G3-G4) | -0.06 | 0.18 | 0.35 | 0.73 |
| Phonological preview benefit * Grade (G4-G5) | 0.09 | 0.18 | 0.47 | 0.64 |
| Phonological preview benefit * Grade (G5-Adult) | -0.32 | 0.18 | 1.81 | 0.07 |

1. **The semantic preview benefit and preview cost for Experiment 3**

As shown in Table S3, on the target character of Experiment 3, there were no reliable effects across FFD, SFD and GD (|*ts*| < 0.81, *ps* > 0.05), though there was a numerical tendency towards shorter reading times on these measures for the target character with grade. Turning to go-past time, there was no significant difference between second and third graders (*b* = -0.08, *SE* = 0.07, *t* = -1.08, *p* = 0.28), but go-past times were significantly longer for third than fourth graders (*b* = -0.15, *SE* = 0.07, *t* = -2.15, *p* = 0.03). This difference was not statistically robust between the fourth than fifth graders (*b* = 0.07, *SE* = 0.07, *t* = 0.96, *p* = 0.34), and it only approached significance for fifth graders compared with adults (*b* = -0.13, *SE* = 0.07, *t* = -1.88, *p* = 0.06). The total reading times for the target character were numerically longer for second grade students compared with third grade students (*b* = -0.10, *SE* = 0.06, *t* = -1.70, *p* = 0.09). There were significantly longer total reading times for third graders than for fourth graders (*b* = -0.14, *SE* = 0.06, *t* = -2.25, *p* = 0.03), and no difference between fourth and fifth graders (*b* = 0.04, *SE* = 0.06, *t* = 0.57, *p* = 0.57). Total reading times for fifth graders were significantly longer than for adults (*b* = -0.18, *SE* = 0.06, *t* = -2.82, *p* < 0.01).

As to the skipping probability, there was no significant difference between second and third graders (*b* = -0.05, *SE* = 0.21, *z* = -0.22, *p* = 0.83), and numerically fewer skips for third than fourth graders (*b* = 0.40, *SE* = 0.21, *z* = 1.92, *p* = 0.05). Also, somewhat surprisingly, the probability of skipping the target was actually numerically greater for the fourth grade than for the fifth grade participants (*b* = -0.41, *SE* = 0.21, *z* = -2.00, *p* = 0.05), whilst the difference between fifth grade students and the adult readers was not significant (*b* = 0.10, *SE* = 0.21, *z* = 0.50, *p* = 0.62). We note the very high level of consistency in the developmental trends that we expected to occur. These effects appeared directionally for almost all the measures across all of the participant groups and these were very comparable across Experiments 1, 2 and 3. Thus, we show clear developmental differences in the direction we predicted.

Next we turn to the main effects of preview. Across the measures of FFD, SFD, GD, go-past time, there were main effects of preview (*|ts|* > 3.06, *ps* < 0.01), with the shortest times under the identical preview condition and the longest times under the unrelated condition. Total reading times for the identical condition were significantly shorter than for the related conditions (i.e., there was a preview cost) (*b* = 0.12, *SE* = 0.02, *t* = 6.75, *p* < 0.001), while the difference between the semantically related and unrelated conditions (the semantic preview benefit main effect) missed significance (*b* = 0.03, *SE* = 0.02, *t* = 1.68, *p* = 0.09). On skipping probability, there was no significant difference between the identical and the related conditions (i.e., preview cost) (*b* = -0.03, *SE* = 0.06, *z* = -0.56, *p* = 0.58). Skipping probability was significantly higher in the related than the unrelated condition (*b* = -0.17, *SE* = 0.06, *z* = -2.86, *p* < 0.01), reflecting preview benefit.

In relation to interactive effects, there was an interaction between preview benefit (semantically related – unrelated preview) across grades for gaze duration, such that the semantic preview benefit effect was robust for third grade students, but not so for second grade students (*b* = 0.11, *SE* = 0.05, *t* = 2.07, *p* = 0.04). Preview benefit effects were comparable (i.e., not significantly different) for third, fourth and fifth grade students as well as adults for gaze duration (*|ts|* < 0.57, *ps* > 0.05). Although the interaction for total reading time was not robust, the pattern of effects reflected preview benefit for adult readers compared with fifth grade students (*b* = 0.10, *SE* = 0.06, *t* = 1.75, *p* = 0.08). There were no counterpart differences between the second, third, fourth and fifth grade students (*|ts|* < 1.27, *ps* > 0.05).

Table S3. Results of Linear Mixed Effects on the Target Across Conditions

|  | *β* | *SE* | \|*t*\| or \|*z*\| | *p* |
| --- | --- | --- | --- | --- |
| **First fixation** |  |  |  |  |
| Intercept | 5.55 | 0.01 | 370.39 | < 0.001 |
| Grade 2 vs. Grade 3 | -0.02 | 0.04 | 0.47 | 0.64 |
| Grade 3 vs. Grade 4 | -0.02 | 0.04 | 0.46 | 0.65 |
| Grade 4 vs. Grade 5 | -0.02 | 0.04 | 0.41 | 0.68 |
| Grade 5 vs. Adult | -0.01 | 0.04 | 0.14 | 0.89 |
| Preview cost | 0.08 | 0.02 | 5.20 | < 0.001 |
| Semantic preview benefit | 0.05 | 0.02 | 3.07 | < 0.01 |
| Preview cost * Grade (G2-G3) | -0.06 | 0.05 | 1.32 | 0.19 |
| Preview cost * Grade (G3-G4) | 0.04 | 0.05 | 0.82 | 0.41 |
| Preview cost * Grade (G4-G5) | -0.00 | 0.05 | 0.02 | 0.99 |
| Preview cost * Grade (G5-Adult) | -0.03 | 0.05 | 0.54 | 0.59 |
| Semantic preview benefit * Grade (G2-G3) | 0.07 | 0.05 | 1.45 | 0.15 |
| Semantic preview benefit * Grade (G3-G4) | 0.00 | 0.05 | 0.07 | 0.95 |
| Semantic preview benefit * Grade (G4-G5) | 0.05 | 0.05 | 0.97 | 0.33 |
| Semantic preview benefit * Grade (G5-Adult) | -0.00 | 0.05 | 0.10 | 0.92 |
| **Single fixation** |  |  |  |  |
| Intercept | 5.54 | 0.02 | 367.43 | < 0.001 |
| Grade 2 vs. Grade 3 | -0.02 | 0.04 | 0.55 | 0.58 |
| Grade 3 vs. Grade 4 | -0.01 | 0.05 | 0.14 | 0.89 |
| Grade 4 vs. Grade 5 | -0.01 | 0.05 | 0.31 | 0.75 |
| Grade 5 vs. Adult | -0.01 | 0.04 | 0.26 | 0.79 |
| Preview cost | 0.07 | 0.02 | 4.50 | < 0.001 |
| Semantic preview benefit | 0.06 | 0.02 | 3.55 | < 0.001 |
| Preview cost * Grade (G2-G3) | -0.09 | 0.05 | 1.71 | 0.09 |
| Preview cost * Grade (G3-G4) | 0.04 | 0.05 | 0.75 | 0.45 |
| Preview cost * Grade (G4-G5) | -0.00 | 0.05 | 0.02 | 0.99 |
| Preview cost * Grade (G5-Adult) | -0.04 | 0.05 | 0.80 | 0.42 |
| Semantic preview benefit * Grade (G2-G3) | 0.08 | 0.05 | 1.51 | 0.13 |
| Semantic preview benefit * Grade (G3-G4) | 0.02 | 0.05 | 0.30 | 0.77 |
| Semantic preview benefit * Grade (G4-G5) | 0.06 | 0.05 | 1.26 | 0.21 |
| Semantic preview benefit * Grade (G5-Adult) | -0.00 | 0.05 | 0.03 | 0.97 |
| **Gaze duration** |  |  |  |  |
| Intercept | 5.60 | 0.02 | 320.19 | < 0.001 |
| Grade 2 vs. Grade 3 | -0.04 | 0.05 | 0.80 | 0.42 |
| Grade 3 vs. Grade 4 | -0.04 | 0.05 | 0.70 | 0.48 |
| Grade 4 vs. Grade 5 | -0.01 | 0.05 | 0.10 | 0.92 |
| Grade 5 vs. Adult | -0.02 | 0.05 | 0.34 | 0.74 |
| Preview cost | 0.08 | 0.02 | 4.84 | < 0.001 |
| Semantic preview benefit | 0.08 | 0.02 | 4.72 | < 0.001 |
| Preview cost * Grade (G2-G3) | -0.10 | 0.05 | 1.97 | 0.05 |
| Preview cost * Grade (G3-G4) | 0.03 | 0.06 | 0.58 | 0.56 |
| Preview cost * Grade (G4-G5) | 0.00 | 0.06 | 0.07 | 0.94 |
| Preview cost * Grade (G5-Adult) | -0.00 | 0.05 | 0.09 | 0.93 |
| Semantic preview benefit * Grade (G2-G3) | 0.11 | 0.05 | 2.07 | 0.04 |
| Semantic preview benefit * Grade (G3-G4) | 0.02 | 0.05 | 0.35 | 0.73 |
| Semantic preview benefit * Grade (G4-G5) | 0.00 | 0.05 | 0.04 | 0.97 |
| Semantic preview benefit * Grade (G5-Adult) | 0.03 | 0.05 | 0.56 | 0.57 |
| **Go-past duration** |  |  |  |  |
| Intercept | 5.87 | 0.03 | 224.30 | < 0.001 |
| Grade 2 vs. Grade 3 | -0.08 | 0.07 | 1.08 | 0.28 |
| Grade 3 vs. Grade 4 | -0.15 | 0.07 | 2.15 | 0.03 |
| Grade 4 vs. Grade 5 | 0.07 | 0.07 | 0.96 | 0.34 |
| Grade 5 vs. Adult | -0.13 | 0.07 | 1.88 | 0.06 |
| Preview cost | 0.11 | 0.02 | 4.51 | < 0.001 |
| Semantic preview benefit | 0.12 | 0.02 | 4.77 | < 0.001 |
| Preview cost * Grade (G2-G3) | -0.09 | 0.08 | 1.21 | 0.23 |
| Preview cost * Grade (G3-G4) | 0.02 | 0.08 | 0.27 | 0.79 |
| Preview cost * Grade (G4-G5) | -0.03 | 0.08 | 0.35 | 0.73 |
| Preview cost * Grade (G5-Adult) | 0.02 | 0.08 | 0.21 | 0.84 |
| Semantic preview benefit * Grade (G2-G3) | 0.06 | 0.07 | 0.85 | 0.40 |
| Semantic preview benefit * Grade (G3-G4) | 0.05 | 0.08 | 0.61 | 0.54 |
| Semantic preview benefit * Grade (G4-G5) | 0.01 | 0.08 | 0.16 | 0.87 |
| Semantic preview benefit * Grade (G5-Adult) | -0.02 | 0.08 | 0.26 | 0.80 |
| **Total duration** |  |  |  |  |
| Intercept | 5.87 | 0.02 | 252.98 | < 0.001 |
| Grade 2 vs. Grade 3 | -0.10 | 0.06 | 1.70 | 0.09 |
| Grade 3 vs. Grade 4 | -0.14 | 0.06 | 2.25 | 0.03 |
| Grade 4 vs. Grade 5 | 0.04 | 0.06 | 0.57 | 0.57 |
| Grade 5 vs. Adult | -0.18 | 0.06 | 2.82 | < 0.01 |
| Preview cost | 0.12 | 0.02 | 6.75 | < 0.001 |
| Semantic preview benefit | 0.03 | 0.02 | 1.68 | 0.09 |
| Preview cost * Grade (G2-G3) | -0.02 | 0.05 | 0.44 | 0.66 |
| Preview cost * Grade (G3-G4) | 0.06 | 0.06 | 1.08 | 0.28 |
| Preview cost * Grade (G4-G5) | 0.01 | 0.06 | 0.26 | 0.80 |
| Preview cost * Grade (G5-Adult) | -0.03 | 0.06 | 0.50 | 0.62 |
| Semantic preview benefit * Grade (G2-G3) | 0.07 | 0.05 | 1.26 | 0.21 |
| Semantic preview benefit * Grade (G3-G4) | -0.01 | 0.05 | 0.26 | 0.79 |
| Semantic preview benefit * Grade (G4-G5) | -0.04 | 0.05 | 0.68 | 0.50 |
| Semantic preview benefit * Grade (G5-Adult) | 0.10 | 0.06 | 1.75 | 0.08 |
| **Skipping probability** |  |  |  |  |
| Intercept | 0.04 | 0.08 | 0.57 | 0.57 |
| Grade 2 vs. Grade 3 | -0.05 | 0.21 | 0.22 | 0.83 |
| Grade 3 vs. Grade 4 | 0.40 | 0.21 | 1.92 | 0.05 |
| Grade 4 vs. Grade 5 | -0.41 | 0.21 | 2.00 | 0.05 |
| Grade 5 vs. Adult | 0.10 | 0.21 | 0.50 | 0.62 |
| Preview cost | -0.03 | 0.06 | 0.56 | 0.58 |
| Semantic preview benefit | -0.17 | 0.06 | 2.86 | < 0.01 |
| Preview cost * Grade (G2-G3) | 0.00 | 0.18 | 0.03 | 0.98 |
| Preview cost * Grade (G3-G4) | -0.03 | 0.18 | 0.16 | 0.88 |
| Preview cost * Grade (G4-G5) | 0.22 | 0.18 | 1.21 | 0.23 |
| Preview cost * Grade (G5-Adult) | 0.13 | 0.18 | 0.72 | 0.47 |
| Semantic preview benefit * Grade (G2-G3) | -0.04 | 0.18 | 0.23 | 0.82 |
| Semantic preview benefit * Grade (G3-G4) | -0.11 | 0.18 | 0.59 | 0.56 |
| Semantic preview benefit * Grade (G4-G5) | -0.20 | 0.18 | 1.11 | 0.27 |
| Semantic preview benefit * Grade (G5-Adult) | 0.02 | 0.19 | 0.10 | 0.92 |

1. **LMM analyses on the target when launch site was included as a continuous variable in Experiments 1-3.**

We analysed launch site as a continuous independent variable in Experiments 1, 2 and 3. The results showed that there was very little modulatory effect of this variable in the analyses for each experiment. For Experiment 1, there was one two-way interaction and one three-way interaction out of 18 analyses – these effects do not appear to be large or systematic. For Experiment 2 there was a significant interaction between phonological preview benefit and launch site for Grades 2 and 3, however, these effects are clearly meaningless because the second and third graders in this experiment did not actually show phonological preview effects. The interaction between phonological preview benefit and the launch site for Grade 4 in single fixation duration (but no other measures) was marginal (*b* = 0.04, *SE* = 0.02, *t* = 1.92, *p* = 0.05). No other effects were significant for Experiment 2. Finally, for Experiment 3 there were no significant effects (see Tables S4-6). We interpret the full set of analyses (54 statistical tests) as demonstrating that there were no meaningful effects of launch site on the preview benefit effects we observed across all three experiments.

Table S4 LMM Analyses on the Target When Launch Site Was Included as a Continuous Variable（Experiment 1, Orthographic Preview Benefit）

| **Factor** | **FFD** | | | **GD** | | | ***SFD*** | | |
| --- | --- | --- | --- | --- | --- | --- | --- | --- | --- |
|  | ***β*** | ***SE*** | **t** | ***β*** | ***SE*** | **t** | ***β*** | ***SE*** | **t** |
| **Intercept** | 5.48 | 0.01 | **401.16** | 5.52 | 0.02 | **362.53** | 5.48 | 0.01 | **389.22** |
| **preview** |  |  |  |  |  |  |  |  |  |
| Launch site | -0.02 | 0.004 | **-5.18** | -0.03 | 0.005 | **-6.45** | -0.02 | 0.005 | **-5.31** |
| **interactions** |  |  |  |  |  |  |  |  |  |
| Preview cost * Launch site(G2) | -0.02 | 0.02 | -1.06 | -0.05 | 0.02 | **-2.06** | -0.03 | 0.01 | -1.26 |
| Preview cost * Launch site(G3) | -0.010 | 0.02 | -0.57 | -0.01 | 0.02 | -0.54 | -0.01 | 0.02 | -0.48 |
| Preview cost * Launch site(G4) | -0.03 | 0.02 | -1.23 | -0.02 | 0.02 | -1.03 | -0.03 | 0.02 | -1.62 |
| Preview cost * Launch site(G5) | -0.03 | 0.02 | -1.44 | -0.04 | 0.02 | -1.63 | -0.03 | 0.02 | -1.33 |
| Preview cost * Launch site(Adult) | -0.004 | 0.03 | -0.14 | -0.01 | 0.03 | -0.39 | -0.003 | 0.03 | -0.11 |
| Orthographic preview benefit * Launch site(G2) | 0.02 | 0.02 | 1.10 | 0.05 | 0.02 | 1.88 | 0.03 | 0.02 | 1.22 |
| Orthographic preview benefit * Launch site(G3) | -0.008 | 0.02 | -0.48 | -0.02 | 0.02 | -0.98 | -0.01 | 0.02 | -0.79 |
| Orthographic preview benefit * Launch site(G4) | 0.004 | 0.02 | 0.24 | 0.003 | 0.02 | 0.18 | 0.01 | 0.02 | 0.38 |
| Orthographic preview benefit * Launch site(G5) | 0.01 | 0.02 | 0.68 | 0.01 | 0.02 | 0.41 | 0.01 | 0.02 | 0.51 |
| Orthographic preview benefit * Launch site(Adult) | 0.01 | 0.02 | 0.36 | -0.01 | 0.03 | -0.21 | 0.004 | 0.03 | 0.16 |
| Preview cost * Launch site * Grade (G2-G3) | 0.009 | 0.02 | 0.36 | 0.03 | 0.03 | 1.24 | 0.01 | 0.03 | 0.58 |
| Preview cost * Launch site * Grade (G3-G4) | -0.02 | 0.03 | -0.67 | -0.01 | 0.03 | -0.45 | -0.03 | 0.03 | -1.04 |
| Preview cost * Launch site * Grade (G4-G5) | -0.007 | 0.03 | -0.21 | -0.02 | 0.03 | -0.53 | 0.003 | 0.03 | 0.10 |
| Preview cost * Launch site * Grade (G5-Adult) | 0.03 | 0.04 | 0.79 | 0.03 | 0.04 | 0.78 | 0.03 | 0.04 | 0.75 |
| Orthographic preview benefit * Launch site * Grade (G2-G3) | -0.03 | 0.03 | -1.16 | -0.06 | 0.03 | **-2.25** | -0.04 | 0.03 | -1.45 |
| Orthographic preview benefit * Launch site * Grade (G3-G4) | 0.01 | 0.02 | 0.60 | 0.02 | 0.03 | 0.91 | 0.02 | 0.02 | 0.94 |
| Orthographic preview benefit * Launch site * Grade (G4-G5) | 0.008 | 0.03 | 0.31 | 0.005 | 0.03 | 0.18 | 0.002 | 0.03 | 0.08 |
| Orthographic preview benefit * Launch site * Grade (G5-Adult) | -0.005 | 0.03 | -0.14 | -0.02 | 0.04 | -0.42 | -0.006 | 0.03 | -0.16 |

Note: The Identical-Orthographically related differences represent the cost to processing of having an orthographically related preview relative to an identical preview. The Unrelated-Orthographically related differences represent the orthographic preview benefit associated with having an orthographically related preview relative to an unrelated preview.

Significant terms are marked in bold, and marginally significant items are underlined.

Table S5 LMM Analyses on the Target When Launch Site Was Included as a Continuous Variable（Experiment 2, Phonological Preview Benefit）

| **Factor** | **FFD** | | | **GD** | | | ***SFD*** | | |
| --- | --- | --- | --- | --- | --- | --- | --- | --- | --- |
|  | ***β*** | ***SE*** | **t** | ***β*** | ***SE*** | **t** | ***β*** | ***SE*** | **t** |
| **Intercept** | 5.54 | 0.01 | **398.85** | 5.60 | 0.02 | **362.47** | 5.54 | 0.01 | **383.87** |
| **preview** |  |  |  |  |  |  |  |  |  |
| Launch site | -0.03 | 0.005 | **-6.66** | -0.04 | 0.005 | **-7.84** | -0.03 | 0.005 | **-6.15** |
| **interactions** |  |  |  |  |  |  |  |  |  |
| Preview cost * Launch site(G2) | 0.003 | 0.02 | 0.12 | -0.01 | 0.03 | -0.45 | -0.004 | 0.02 | -0.18 |
| Preview cost * Launch site(G3) | -0.03 | 0.02 | -1.24 | -0.03 | 0.03 | -1.31 | -0.04 | 0.02 | -1.55 |
| Preview cost * Launch site(G4) | -0.02 | 0.02 | -1.08 | -0.04 | 0.02 | -1.47 | -0.04 | 0.02 | -1.59 |
| Preview cost * Launch site(G5) | -0.04 | 0.02 | -1.87 | -0.05 | 0.02 | **-2.20** | -0.04 | 0.02 | -1.93 |
| Preview cost * Launch site(Adult) | -0.01 | 0.03 | -0.37 | -0.02 | 0.03 | -0.64 | -0.01 | 0.03 | -0.28 |
| Phonological preview benefit * Launch site(G2) | -0.06 | 0.02 | **-2.74** | -0.04 | 0.03 | -1.73 | -0.05 | 0.02 | **-2.19** |
| Phonological preview benefit * Launch site(G3) | 0.05 | 0.02 | **2.38** | 0.05 | 0.02 | 1.93 | 0.07 | 0.02 | **3.02** |
| Phonological preview benefit * Launch site(G4) | 0.04 | 0.02 | 1.88 | 0.03 | 0.02 | 1.29 | 0.04 | 0.02 | 1.92 |
| Phonological preview benefit * Launch site(G5) | -0.004 | 0.02 | -0.19 | -0.01 | 0.02 | -0.54 | 0.01 | 0.02 | 0.20 |
| Phonological preview benefit * Launch site(Adult) | -0.01 | 0.03 | -0.24 | 0.002 | 0.03 | 0.06 | -0.01 | 0.03 | -0.32 |
| Preview cost * Launch site * Grade (G2-G3) | -0.03 | 0.03 | -0.96 | -0.02 | 0.03 | -0.65 | -0.03 | 0.03 | -0.97 |
| Preview cost * Launch site * Grade (G3-G4) | 0.006 | 0.03 | 0.20 | 0.002 | 0.03 | 0.07 | 0.003 | 0.03 | 0.08 |
| Preview cost * Launch site * Grade (G4-G5) | -0.02 | 0.03 | -0.49 | -0.02 | 0.04 | -0.52 | -0.006 | 0.03 | -0.18 |
| Preview cost * Launch site * Grade (G5-Adult) | 0.03 | 0.04 | 0.71 | 0.03 | 0.04 | 0.74 | 0.03 | 0.04 | 0.85 |
| Phonological preview benefit * Launch site * Grade (G2-G3) | 0.11 | 0.03 | **3.78** | 0.09 | 0.03 | **2.67** | 0.12 | 0.03 | **3.88** |
| Phonological preview benefit * Launch site * Grade (G3-G4) | -0.009 | 0.03 | -0.30 | -0.02 | 0.03 | -0.46 | -0.02 | 0.03 | -0.79 |
| Phonological preview benefit * Launch site * Grade (G4-G5) | -0.05 | 0.03 | -1.50 | -0.04 | 0.04 | -1.27 | -0.04 | 0.03 | -1.25 |
| Phonological preview benefit * Launch site * Grade (G5-Adult) | 0.006 | 0.04 | 0.15 | 0.02 | 0.04 | 0.57 | -0.005 | 0.04 | -0.15 |

Note: The Identical-Phonologically related differences represent the cost to processing of having a phonologically related preview relative to an identical preview. The Unrelated-Phonologically related differences represent the phonological preview benefit associated with having a phonologically related preview relative to an unrelated preview. Significant terms are marked in bold, and marginally significant items are underlined.

Table S6 LMM Analyses on the Target When Launch Site Was Included as a Continuous Variable（Experiment 3, Semantic Preview Benefit）

| **Factor** | **FFD** | | | **GD** | | | ***SFD*** | | |
| --- | --- | --- | --- | --- | --- | --- | --- | --- | --- |
|  | ***β*** | ***SE*** | **t** | ***β*** | ***SE*** | **t** | ***β*** | ***SE*** | **t** |
| **Intercept** | 5.55 | 0.01 | **399.36** | 5.61 | 0.02 | **351.94** | 5.48 | 0.01 | **389.22** |
| **preview** |  |  |  |  |  |  |  |  |  |
| Launch site | -0.03 | 0.005 | **2.80** | -0.03 | 0.06 | **-5.81** | -0.02 | 0.004 | **-5.31** |
| **interactions** |  |  |  |  |  |  |  |  |  |
| Preview cost * Launch site(G2) | -0.002 | 0.02 | -0.13 | -0.01 | 0.02 | -0.36 | -0.03 | 0.02 | -1.26 |
| Preview cost * Launch site(G3) | 0.01 | 0.02 | 0.56 | -0.0001 | 0.03 | -0.004 | -0.01 | 0.02 | -0.48 |
| Preview cost * Launch site(G4) | -0.007 | 0.02 | -0.35 | -0.02 | 0.02 | -0.68 | -0.03 | 0.02 | -1.62 |
| Preview cost * Launch site(G5) | -0.04 | 0.02 | -1.51 | -0.04 | 0.03 | -1.68 | -0.03 | 0.02 | -1.33 |
| Preview cost * Launch site(Adult) | -0.002 | 0.03 | -0.08 | -0.01 | 0.03 | -0.23 | -0.002 | 0.03 | -0.11 |
| Semantic preview benefit * Launch site(G2) | -0.01 | 0.02 | -0.39 | -0.01 | 0.02 | -0.50 | 0.03 | 0.02 | 1.22 |
| Semantic preview benefit * Launch site(G3) | -0.03 | 0.02 | -1.16 | -0.04 | 0.03 | -1.61 | -0.01 | 0.02 | -0.79 |
| Semantic preview benefit * Launch site(G4) | -0.004 | 0.02 | -0.26 | -0.01 | 0.02 | -0.62 | 0.01 | 0.02 | 0.38 |
| Semantic preview benefit * Launch site(G5) | 0.01 | 0.02 | 0.47 | 0.03 | 0.03 | 1.16 | 0.01 | 0.02 | 0.51 |
| Semantic preview benefit * Launch site(Adult) | -0.02 | 0.03 | -0.76 | -0.04 | 0.03 | -1.31 | 0.004 | 0.03 | 0.16 |
| Preview cost * Launch site * Grade (G2-G3) | 0.02 | 0.03 | 0.61 | 0.01 | 0.03 | 0.37 | 0.01 | 0.03 | 0.58 |
| Preview cost * Launch site * Grade (G3-G4) | -0.02 | 0.03 | -0.57 | -0.02 | 0.04 | -0.43 | -0.03 | 0.03 | -1.04 |
| Preview cost * Launch site * Grade (G4-G5) | -0.03 | 0.03 | -0.90 | -0.03 | 0.04 | -0.82 | 0.003 | 0.03 | 0.10 |
| Preview cost * Launch site * Grade (G5-Adult) | 0.03 | 0.04 | 0.83 | 0.04 | 0.04 | 0.88 | 0.03 | 0.04 | 0.75 |
| Semantic preview benefit * Launch site * Grade (G2-G3) | -0.02 | 0.03 | -0.66 | -0.03 | -0.03 | -1.0 | -0.04 | 0.03 | -1.45 |
| Semantic preview benefit * Launch site * Grade (G3-G4) | 0.02 | 0.03 | 0.64 | 0.03 | 0.04 | 0.80 | 0.02 | 0.02 | 0.94 |
| Semantic preview benefit * Launch site * Grade (G4-G5) | 0.02 | 0.03 | 0.51 | 0.04 | 0.03 | 1.29 | 0.002 | 0.03 | 0.08 |
| Semantic preview benefit * Launch site * Grade (G5-Adult) | -0.03 | 0.04 | -0.76 | -0.06 | 0.04 | -1.60 | -0.006 | 0.03 | -0.16 |

Note: The Identical-Semantically related differences represent the cost to processing of having a semantically related preview relative to an identical preview. The Unrelated-Semantically related differences represent the semantic preview benefit associated with having a semantically related preview relative to an unrelated preview.

Significant terms are marked in bold, and marginally significant items are underlined
